# Tyrosine kinases in KMT2A/MLL-rearranged acute leukemias as potential therapeutic targets to overcome cancer drug resistance

**DOI:** 10.20517/cdr.2022.78

**Published:** 2022-10-09

**Authors:** Fatih M. Uckun, Sanjive Qazi

**Affiliations:** Ares Pharmaceuticals, St. Paul, MN 55110, USA.

**Keywords:** AML, ALL, MLL gene, tyrosine kinase, Leukemia

## Abstract

**Aim:** The main goal of this study was to elucidate at the transcript level the tyrosine kinase expression profiles of primary leukemia cells from mixed lineage leukemia 1 gene rearranged (KMT2A/MLL-R^+^) acute myeloid leukemia (AML) and acute lymphoblastic leukemia (ALL) patients.

**Methods: **We evaluated protein tyrosine kinase (PTK) gene expression profiles of primary leukemic cells in KMT2A/MLL-R^+^ AML and ALL patients using publicly available archived datasets.

**Results: **Our studies provided unprecedented evidence that the genetic signatures of KMT2A/MLL-R^+^ AML and ALL cells are characterized by transcript-level overexpression of specific PTK. In infants, children and adults with KMT2A/MLL-R^+^ ALL, as well as pediatric patients with KMT2A/MLL-R^+^ AML, the gene expression levels for FLT3, BTK, SYK, JAK2/JAK3, as well as several SRC family PTK were differentially amplified. In adults with KMT2A/MLL-R^+^ AML, the gene expression levels for SYK, JAK family kinase TYK2, and the SRC family kinases FGR and HCK were differentially amplified.

**Conclusion: **These results provide new insights regarding the clinical potential of small molecule inhibitors of these PTK, many of which are already FDA/EMA-approved for other indications, as components of innovative multi-modality treatment platforms against KMT2A/MLL-R^+^ acute leukemias.

## INTRODUCTION

The lysine [K]-methyltransferase 2A (KMT2A)/mixed-lineage leukemia 1 (KMT2A/MLL) gene on chromosome 11 encodes a 431-kDa protein involved in the regulation of transcription^[1]^. Rearrangements (r) of the KMT2A/MLL gene have been reported in acute myeloid leukemia (AML) and acute lymphoblastic leukemia (ALL)^[2-14]^. AML and ALL patients with KMT2A/MLL-R^+^ leukemia have a poor prognosis with disappointing event-free survival (EFS) and overall survival (OS) outcomes on contemporary treatment regimens due to relapses caused by cancer drug-resistant clones^[1-19]^. Therefore, more effective frontline as well as salvage treatments for MLL-R^+^ acute leukemias are urgently needed. 

The main goal of the present study was to evaluate the protein tyrosine kinase (PTK) profiles of primary leukemic cells in patients with KMT2A/MLL-R^+^ AML and ALL as potential therapeutic targets to overcome cancer drug resistance. We compared the transcriptomes of primary leukemic cells from KMT2A/MLL-R^+^ *vs*. KMT2A/MLL-R^-^ AML and ALL patients with an emphasis on the relative gene expression levels of for 21 PTK, including ERBB1, FGR, FLT3, FYN, HCK, JAK2, LCK, LYN, MERTK, SRC, BLK, BMX, BTK, ERBB2, ERBB3, JAK1, JAK3, PTK2, SYK, TEC, TYK2. Our studies provided unprecedented evidence that the genetic signatures of KMT2A/MLL-R^+^ AML and ALL cells are characterized by transcript-level overexpression of specific PTK. In infants, children and adults with KMT2A/MLL-R^+^ ALL, as well as pediatric patients with KMT2A/MLL-R^+^ AML, the gene expression levels for FLT3, BTK, SYK, JAK2/JAK3, as well as several SRC family PTK were differentially amplified. In adults with KMT2A/MLL-R^+^ AML, the gene expression levels for SYK, JAK family member TYK2, and the SRC family PTK HCK and FGR were differentially amplified. These results provide new insights regarding the clinical potential of small molecule inhibitors of these PTK, many of which are already FDA/EMA-approved for other indications, as components of innovative multi-modality treatment platforms against KMT2A/MLL-R^+^ acute leukemias. 

## METHODS

### Statistical methods for gene chip normalization for ALL and AML samples 

A recently published working database, including data on primary leukemia cells from 201 adult patients with B-ALL (GSE13159), 119 pediatric patients with B-ALL (GSE11877 and GSE13351) and 97 infants with B-ALL (GSE68720), as well as 74 normal/non-leukemic control bone marrow samples (GSE13159), was built with previously archived datasets from the NCBI repository and used in our comparative gene expression analyses, as previously described in detail^[20]^. We also used a working database derived from archived datasets including data on primary leukemia cells from 542 adult patients with AML (GSE13159), and 279 pediatric patients with AML (GSE19577, GSE17855) along with the 74 normal/non-leukemic control bone marrow samples (GSE13159) in our comparative gene expression analyses, as described^[20]^. Probeset level normalization procedures were used as previously reported^[20]^. 

We also compared the gene expression profiles of primary leukemia cells from FLT3-ITD^+^ pediatric AML patients (*N* = 48 from GSE17855) with those of primary leukemia cells from 189 FLT3-ITD^-^ pediatric AML patients (GSE17855) as well as normal hematopoietic cells from 74 non-leukemic control bone marrow samples (GSE13159). Forty-seven patients from the KMT2A/MLL-R^-^ “other” group of pediatric AML patients were FLT3-ITD^+^, including 18 patients with cytogenetically normal AML, one patient with inv^[16]^ AML, 12 patients with t(15;17) AML, 3 patients with t(8;21), 9 patients with AML and other cytogenetic features, and 4 patients who had AML with unknown cytogenetic features. One pediatric patient with KMT2A/MLL-R^+^ AML was FLT3-ITD^+^. This double mutant (FLT3-ITD/MLL-R) case was removed from the analysis of FLT3-ITD^+^ (*N* = 47; GSE17855) versus KMT2A/MLL-R^+^ pediatric AML patients (*N* = 88; GSE17855, *N* = 46; GSE19577, *N* = 42). 

No pediatric patients with KMT2A/MLL-R^+^ AML harbored *NPM1* or *CEBPA* mutations. Within the KMT2A/MLL-R^-^ (“other”) group of KMT2A/MLL-R^+^ AML pediatric AML patients, 17 harbored *NPM1* mutations (NPM1^+^) and 16 patients had *CEBPA* mutations (CEBPA^+^) (GSE17855). We investigated the gene expression profiles from primary leukemia cells from 71 KMT2A/MLL-R^-^ pediatric AML patients who were FLT3-ITD^+^, NPM1^+^ or CEBPA^+^ (GSE17855; only NPM1^+^ *N* = 10, only CEBPA^+^ *N* = 14, only FLT3-ITD^+^ *N* = 38; 9 cases harbored one or more of the *NPM1*, *CEBPA* or *FLT3-ITD* mutations) versus 88 cases of KMT2A/MLL-R^+^ [GSE17855 (*N* = 46); GSE19577 (*N* = 42)]. One patient with both FLT3-ITD and MLL-R was removed from this comparison.

### Statistical methods for differential gene expression

Our analyses for ALL and AML focused on the expression levels (interrogated with 64 probesets) of the following 21 PTK genes: *ERBB1, FGR, FLT3, FYN, HCK, JAK2, LCK, LYN, MERTK, SRC, BLK, BMX, BTK, ERBB2, ERBB3, JAK1, JAK3, PTK2, SYK, TEC* and* TYK*2. Standard statistical methods, including mixed model ANOVAs, and hierarchical clustering method were employed, as reported^[20-24]^. 

## RESULTS

### Differentially amplified expression of PTK genes in primary leukemic cells from KMT2A/MLL-R^+^ B-ALL patients

We first examined PTK gene expression profiles of primary leukemic cells from infants (*N* = 80), children (*N* = 25) and adults (*N* = 70) with KMT2A/MLL-R^+^ B-ALL *vs*. normal hematopoietic cells from healthy volunteers (*N* = 74). Notably, FMS-like tyrosine kinase 3(FLT3) expression in infant KMT2A/MLL-R^+^ ALL cases was 16.09-fold higher than in normal hematopoietic cells in non-leukemic control bone marrow samples (*P*-value < 1 × 10^-8^) [Supplementary Figure 1; Supplementary Table 1]. The genes for several additional PTK showed augmented expression in KMT2A/MLL-R^+^ infant ALL cells, including TEC, SRC, BLK, JAK2, BTK, PTK (3 probesets), SYK and JAK1 (2 probesets) [Supplementary Figure 1; Supplementary Table 1]. As the second most upregulated gene, the gene for BLK was expressed at a 6.77-fold higher level in infant KMT2A/MLL-R^+^ ALL cells than in normal hematopoietic cells (*P*-value < 1 × 10^-8^) [Supplementary Table 1]. Similarly, FLT3 wasexpressed at a 22.38-fold higher level in pediatric KMT2A/MLL-R^+^ ALL cells than in normal hematopoietic cells (*P*-value < 1 × 10^-8^) [Supplementary Figure 2; Supplementary Table 2]. As in infant leukemia cells, BLK was the second most upregulated gene in pediatric KMT2A/MLL-R^+^ ALL cells showing a 4.38-fold higher expression level than in normal hematopoietic cells (*P*-value < 1 × 10^-8^). The genes for several additional PTK showed augmented expression in pediatric KMT2A/MLL-R^+^ ALL cells, including PTK2, TEC, BTK, and SYK [Supplementary Figure 2; Supplementary Table 2]. Similarly, FLT3 and BLK expression in leukemic cells from adult patients with KMT2A/MLL-R^+^ ALL were 21.00-fold (*P*-value < 1 × 10^-8^) and 21.79-fold (*P*-value < 1 × 10^-8^), respectively, higher than in normal hematopoietic cells [Supplementary Figure 3; Supplementary Table 3]. 

**Figure 1:**
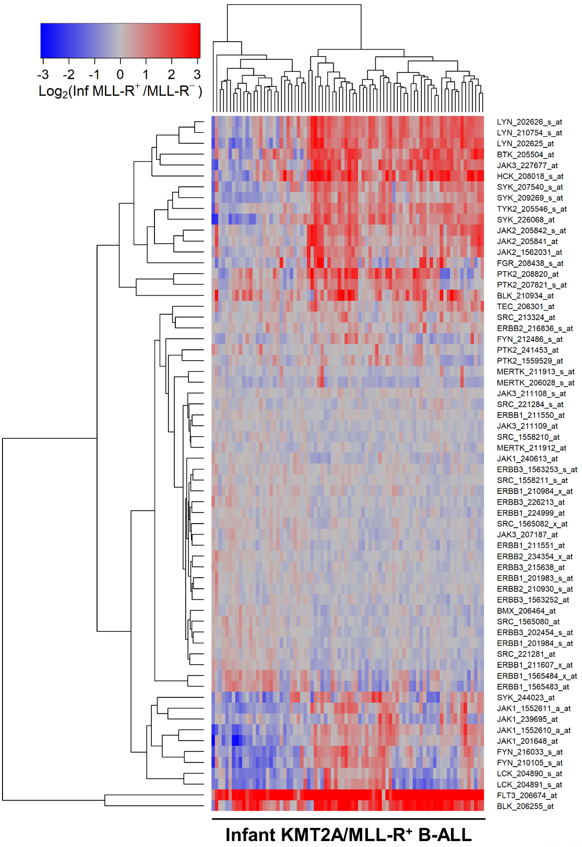
Gene Expression Levels for Tyrosine Kinases in Leukemic Cells from infants with KMT2A/MLL-R^+^ B-ALL *vs.* Other Types of B-ALL without KMT2A/MLL Rearrangements. Probeset level normalized, robust multi-array analysis (RMA) signal values from the archived data for infant ALL (GSE68720) were examined in these comparisons. Infant KMT2A/MLL-R^+^ B-ALL gene partners for KMT2A were AF4 (*N* = 48), ENL (*N* = 16), AF9 (*N* = 6), ASAH3 (*N* = 1), EPS15 (*N* = 3), Unknown (*N* = 6) (GSE68720; Total *N* = 80). The cluster figure displays the expression levels in KMT2A/MLL-R^+^ ALL cells mean centered to the reference group [KMT2A/MLL germline/WT gene (KMT2A/MLL-R-)] represented by log_2_-transformed fold change values (blue to red color indicates under-expression to over-expression respectively in KMT2A/MLL-R^+^ samples). Co-regulated probesets are organized and depicted by dendrograms for both probesets (rows) and patients (columns). The log_2_-transformed RMA values for leukemic cells from 80 infants with KMT2A/MLL-R^+^ B-ALL compared to that from leukemia cells obtained from 17 infants with MLL-germline/WT ALL (MLL-R negative) showed 19 probesets that were upregulated in KMT2A/MLL-R^+^ infant ALL cells. FLT3_206674_at was the most significantly upregulated probeset (Fold Change = 11.23; *P*-value < 10^-8^) followed by BLK_206255_at (Fold Change = 3.98; *P*-value < 10^-8^) and HCK_208018_s_at (Fold Change = 2.46; *P*-value < 10^-8^) [Supplementary Table 4]. Cluster visualization of the mean centered expression values suggested co-regulation of LYN (3 probesets), BTK, JAK3, HCK, SYK (3 probesets), TYK2, JAK2 (3 probesets), FGR, PTK2 (2 probesets) and BLK (2 probesets).

**Figure 2:**
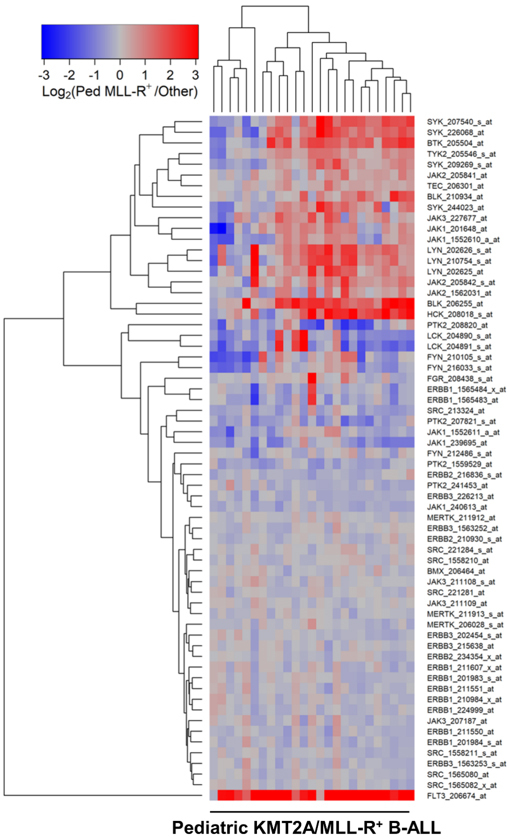
Gene Expression Levels for Tyrosine Kinases in Leukemic Cells from Pediatric Patients with KMT2A/MLL-R^+^ B-ALL *vs*. Other Types of B-ALL without KMT2A/MLL Rearrangements. Probeset level normalized signal values from the archived data sets GSE11877 and GSE13351 were examined in these comparisons. The cluster figure displays the expression levels in KMT2A/MLL-R^+^ ALL cells mean centered to the reference group (other subsets of ALL without KMT2A/MLL rearrangements) represented by log_2_-transformed fold change values (blue to red color indicates under-expression to over-expression respectively in KMT2A/MLL-R^+^ samples). Co-regulated probesets are organized and depicted by dendrograms for both probesets (rows) and patients (columns). Depicted are the differential gene expression changes of log_2_-transformed, robust multi-array analysis (RMA) normalized values for 25 pediatric ALL KMT2A/MLL-R^+^ cases (GSE11877 and GSE13351) compared to 94 non-MLL-R^+^ other samples (GSE11877 and GSE13351) exhibiting 20 dysregulated probesets, of which 14 were upregulated in KMT2A/MLL-R^+^ subset of cases. FLT3_206674_at was the most significantly upregulated probeset (Fold Change = 8.65; *P*-value < 10^-8^) followed by BLK_206255_at (Fold Change = 3.11; *P*-value < 10^-8^) and HCK_208018_s_at (Fold Change = 2.64; *P*-value < 10^-8^) [Supplementary Table 5].

**Figure 3:**
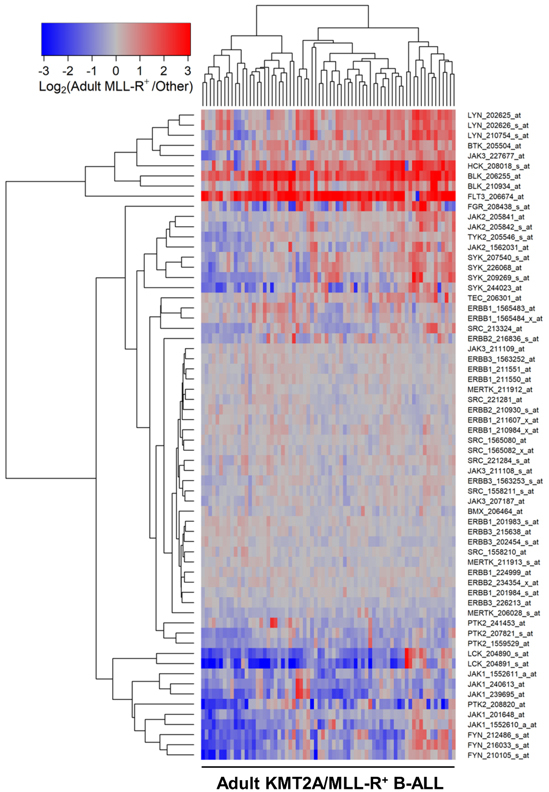
Gene Expression Levels for Tyrosine Kinases in Leukemic Cells from Adult Patients with KMT2A/MLL-R^+^ B-ALL *vs*. Other Types of B-ALL without KMT2A/MLL Rearrangements. Probeset level normalized signal values from the archived data set GSE13159 were examined in these comparisons. The cluster figure displays the expression levels in KMT2A/MLL-R^+^ ALL cells mean centered to the reference group (other types of ALL without KMT2A/MLL Rearrangements) represented by log_2_-transformed fold change values (blue to red color indicates under-expression to over-expression respectively in KMT2A/MLL-R^+^ samples). Co-regulated probesets are organized and depicted by dendrograms for both probesets (rows) and patients (columns). The comparison of the log_2_-transformed RMA values for leukemic cells from 70 adult patients with KMT2A/MLL-R^+^ ALL with the RMA values for leukemic cells from 131 adult patients with other forms of ALL resulted in 25 probesets that were significantly dysregulated, of which 10 were upregulated in KMT2A/MLL-R^+^ subset of cases. FLT3_206674_at was the most significantly upregulated probeset (Fold Change = 5.69; *P*-value < 10^-8^) followed by BLK_206255_at (Fold Change = 3.95; *P*-value < 10^-8^) and HCK_208018_s_at (Fold Change = 2.21; *P*-value < 10^-8^) [Supplementary Table 6]. FLT3 was co-regulated with BLK (2 probesets), HCK and LYN (3 probesets).

We next compared the PTK gene expression profiles of leukemic cells from each KMT2A/MLL-R^+^ subset to the *PTK* gene expression profiles of leukemic cells without KMT2A/MLL rearrangements from the corresponding control ALL patients in the “other” categories (Infants, *N* = 17; Children, *N* = 94; Adults, *N* = 131). In infants [Figure 1; Supplementary Table 4], children [Figure 2; Supplementary Table 5] and adults [Figure 3; Supplementary Table 6] with KMT2A/MLL-R^+^ ALL, the gene expression levels for FLT3, BTK, SYK, JAK2/JAK1, as well as several SRC family PTK, including BLK, were differentially and significantly amplified. In KMT2A/MLL-R^+^ infant ALL cells, FLT3_206674_at was the most significantly upregulated probeset (Fold Change = 11.23; < 10^-8^) followed by BLK_206255_at (Fold Change = 3.98; < 10^-8^) when compared to infant ALL cells with germline/wildtype KMT2A/MLL gene [Figure 1; Supplementary Table 4]. Likewise, in KMT2A/MLL-R^+^ pediatric ALL cells, FLT3_206674_at was the most significantly upregulated probeset (Fold Change = 8.65; *P*-value < 1 × 10^-8^) followed by BLK_206255_at (Fold Change = 3.11; *P*-value < 10^-8^) when compared to pediatric ALL cells without KMT2A/MLL rearrangements [Figure 2; Supplementary Table 5]. Similarly, in KMT2A/MLL-R^+^ adult ALL cells, FLT3_206674_at was the most significantly upregulated probeset (Fold Change = 5.69; *P*-value < 10^-8^) followed by BLK_206255_at (Fold Change = 3.95; *P*-value < 10^-8^) when compared to leukemic cells from adult ALL patients without KMT2A/MLL rearrangements [Figure 3; Supplementary Table 6]. 

### Differentially amplified expression of PTK genes in primary leukemic blasts from KMT2A/MLL-R^+^ AML patients

We examined the PTK gene expression profiles of primary leukemic blasts from 89 children and 38 adults with KMT2A/MLL-R^+^ AML *vs*. normal hematopoietic cells from healthy volunteers (*N* = 74). In comparison to normal hematopoietic cells, KMT2A/MLL-R^+^ pediatric as well as adult AML cells were characterized by amplified expression of FLT3 gene. FLT3_206674_at was the most significantly upregulated probeset (Fold Change in pediatric KMT2A/MLL-R^+^ AML cells = 7.54; *P*-value < 1 × 10^-8^; Fold Change in adult KMT2A/MLL-R^+^ AML cells = 8.28; *P*-value < 1 × 10^-8^) followed by TEC_206301_at (Fold Change in pediatric KMT2A/MLL-R^+^ AML cells = 2.2; *P*-value < 1 × 10^-8^; Fold Change in adult KMT2A/MLL-R^+^ AML cells = 1.73; *P*-value < 1 × 10^-8^) [Supplementary Figures 4 and 5; Supplementary Tables 7 and 8].

**Figure 4:**
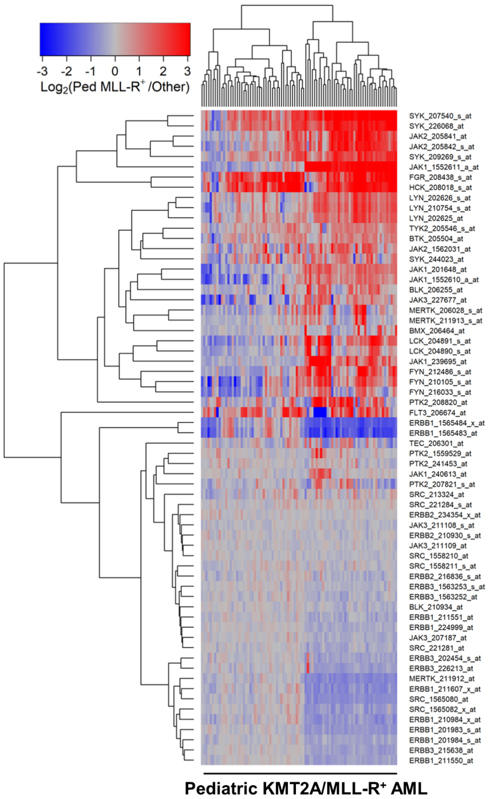
Gene Expression Levels for Tyrosine Kinases in Leukemic Cells from Pediatric Patients with KMT2A/MLL-R^+^ AML *vs*. Other subsets of AML without KMT2A/MLL rearrangements. Probeset level normalized signal values from the archived data sets GSE17855 and GSE19577 were examined in these comparisons. The cluster figure displays the expression levels in KMT2A/MLL-R^+^ AML cells mean centered to the reference group (non-KMT2A/MLL-R^+^ other AML subsets) for log_2_-transformed fold change values (blue to red color indicates under-expression to over-expression respectively in KMT2A/MLL-R^+^ samples). Co-regulated probesets are organized and depicted by dendrograms for both probesets (rows) and patients (columns). Comparing 190 cases of non-KMT2A/MLL-R^+^ other samples (GSE17855) with 89 cases of KMT2A/MLL-R^+^ pediatric AML samples [GSE17855 (*N* = 47) and GSE19577(*N* = 42)] exhibited 38 differentially expressed probesets, of which 25 probesets were significantly upregulated in pediatric KMT2A/MLL-R^+^ subset of cases. FGR_208438_s_at was the most significantly upregulated transcript (Fold Change = 4.31 *P*-value < 10^-8^) followed by SYK_207540_s_at (Fold Change = 4.01; *P*-value < 10^-8^) and HCK_208018_s_at (Fold Change = 3.97; *P*-value < 10^-8^) [Supplementary Table 9]. SYK (3 probesets), JAK2 (2 probesets), JAK1, FGR and HCK formed a cluster of patients with significantly higher expression levels in KMT2A/MLL-R^+^ cases.

**Figure 5:**
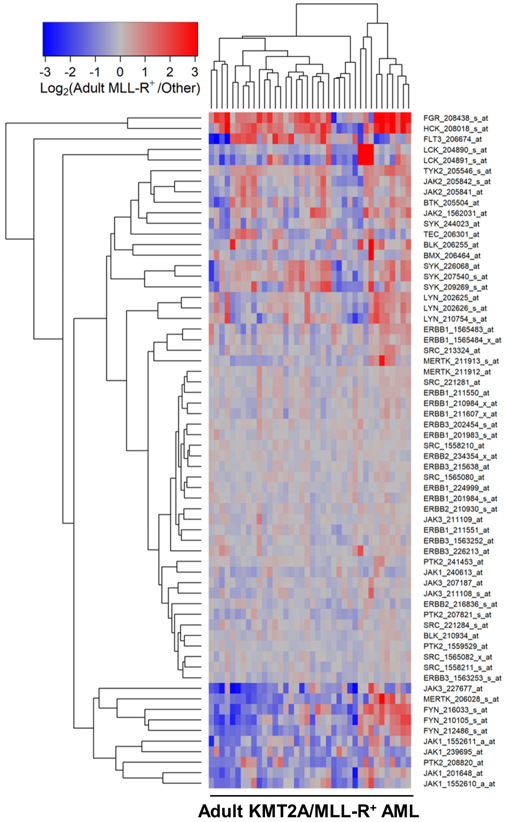
Gene Expression Levels for Tyrosine Kinases in Leukemic Cells from Adult Patients with KMT2A/MLL-R^+^ AML *vs*. Other subsets of AML without KMT2A/MLL rearrangements. Probeset level normalized signal values from the archived data set GSE13159 were examined in these comparisons. The cluster figure displays the expression levels in KMT2A/MLL-R^+^ AML cells mean centered to the reference group (other AML subsets without KMT2A/MLL rearrangements) for log_2_-transformed fold change values (blue to red color indicates under-expression to over-expression respectively in KMT2A/MLL-R^+^ samples). Co-regulated probesets are organized and depicted by dendrograms for both probesets (rows) and patients (columns). Side-by-side comparison of 38 adult MLL-R^+^ AML cases with 504 other non-MLL-R^+^ adult AML cases resulted in 15 dysregulated probesets, of which 6 were upregulated in the adult KMT2A/MLL-R^+^ subset of cases. FGR_208438_s_at was the most significantly upregulated probeset (Fold Change = 2.46; *P*-value < 10^-8^) followed by HCK_208018_s_at (Fold Change = 2.1; *P*-value < 10^-8^) and TYK2_205546_s_at (Fold Change = 1.29; *P*-value = 0.0012) [Supplementary Table 10]. Cluster visualization of the mean centered expression values suggested co-regulation of CD33 with BTK, TYK2 and SYK (3 probesets).

We next compared the PTK gene expression profiles of leukemic cells from pediatric and adult KMT2A/MLL-R^+^ AML cells to the PTK gene expression profiles of AML cells without KMT2A/MLL rearrangements from the corresponding control AML patients in the “other” categories (Children, *N* = 190; Adults, *N* = 504). In pediatric patients with KMT2A/MLL-R^+^ AML, the gene expression levels for FLT3, BTK, SYK, JAK2/JAK3, as well as several SRC family PTK were differentially amplified [Figure 4; Supplementary Table 9]. In adults with KMT2A/MLL-R^+^ AML, the gene expression levels for SYK, JAK family kinase TYK2, and the SRC family kinases FGR and HCK were differentially amplified [Figure 5; Supplementary Table 10]. 

The FLT3-ITD^+^ subset (*N* = 48) among the pediatric AML patients exhibited upregulated FLT3 expression when compared to non-leukemic control samples (*N* = 74) [Supplementary Figure 6; Supplementary Table 11] as well as FLT3-ITD^-^ pediatric AML cases (*N* = 189) [Supplementary Figure 7; Supplementary Table 12]. Comparing 189 cases of FLT3-ITD^-^ samples with 48 cases of FLT3-ITD^+^ pediatric AML samples (GSE17855) exhibited 13 differentially expressed probesets, of which four probesets were significantly upregulated in pediatric FLT3-ITD^+^ cases. FLT3_206674_at was the most significantly upregulated probeset (Fold Change = 1.96; *P*-value < 10^-8^) followed by BTK_205504_at (Fold Change = 1.25; *P*-value = 9.8 × 10^-4^) and TEC_206301_at (Fold Change = 1.23; *P*-value = 0.0025) [Supplementary Figure 7; Supplementary Table 12].

A comparison of the 88 KMT2A/MLL-R^+^ pediatric AML cases with 47 FLT3-ITD^+^ pediatric AML cases showed 31 differentially expressed probesets, of which 24 probesets were significantly upregulated in pediatric KMT2A/MLL-R^+^ cases. FGR_208438_s_at was the most significantly upregulated transcript (Fold Change = 4.40; *P*-value < 10^-8^) followed by SYK_207540_s_at (Fold Change = 3.81; *P*-value < 10^-8^) and JAK1_1552611_a_at (Fold Change = 2.78; *P*-value < 10^-8^). ERBB1_1565483_at was the most significantly downregulated transcript in KMT2A/MLL-R^+^ cases (Fold Change = 0.56; *P*-value < 10^-8^) followed by ERBB1_1565484_x_at (Fold Change = 0.57; *P*-value = 2.5 × 10^-8^) and MERTK_211912_at (Fold Change = 0.73; Pval = 0.0016) [Supplementary Figure 8; Supplementary Table 13]

A comparison of the 88 KMT2A/MLL-R^+^ pediatric AML cases with 71 cases of FLT3-ITD^+^/NPM1^+^/CEBPA^+^ pediatric AML cases exhibited 34 differentially expressed probesets, of which 26 probesets were significantly upregulated in the KMT2A/ MLL-R^+^ subset. FGR_208438_s_at was the most significantly upregulated probeset (Fold Change = 4.31; *P*-value < 10^-8^) followed by SYK_207540_s_at (Fold Change = 3.92; *P*-value < 10^-8^) and HCK_208018_s_at (Fold Change = 2.86; *P*-value < 10^-8^). ERBB1_1565483_at was the most significantly downregulated transcript in KMT2A/MLL-R^+^ cases (Fold Change = 0.55; *P*-value < 10^-8^) followed by ERBB1_1565484_x_at (Fold Change = 0.56; *P*-value < 10^-8^) and MERTK_211912_at (Fold Change = 0.72; *P*-value = 1.5 × 10^-4^) [Supplementary Figure 9; Supplementary Table 14].

## DISCUSSION

The outcome of KMT2A/MLL-R^+^ AML and B-ALL patients, especially those with relapsed or refractory leukemia, is disappointingly poor after contemporary treatments^[3,5-8,10-14]^. New treatment strategies are urgently needed for both KMT2A/MLL-R^+^ AML and KMT2A/MLL-R^+^ ALL. Therefore, a large panel of drugs are being evaluated as potential therapeutic agents against KMT2A/MLL-R^+^ AML and ALL, including inhibitors of Menin-MLL1 interaction^[25-28]^. The present study demonstrates that PTK inhibitors (PTKi), especially inhibitors of FLT3, may have clinical impact potential as therapeutic agents against KMT2A/MLL-R^+^ B-ALL as well as AML. Further, inhibitors of SRC family PTK may be clinically useful against KMT2A/MLL-R^+^ B-ALL and inhibitors of BTK, SYK, and JAK family PTK may be clinically useful against KMT2A/MLL-R^+^ AML. If our observations in this small series are confirmed in a larger series of leukemia patients, preferably with corresponding proteomics/phosphoproteomics data, proof-of-concept studies with FDA-approved PTKi would be warranted to further evaluate the clinical potential of PTK targeting in KMT2A/MLL-R^+^ ALL and AML. 

PTK play a critical role in normal lymphohematopoiesis, and they have also been implicated as leukemogenic oncoproteins in the development of acute and chronic leukemias^[29-31]^. The incorporation of PTKi into the standard of care has caused a paradigm shift in the treatment of CML (PTKi: ABL1 and SRC inhibitors), CLL (PTKi: BTK inhibitors), Ph^+^ B-ALL (PTKi: ABL1 and SRC inhibitors), and AML with *FLT3* mutations (PTKi: FLT3 inhibitors)^[29,30]^. There is growing consensus regarding their evolving role in Ph-like B-ALL (PTKi: ABL1, SRC, TRK, FLT3 and JAK inhibitors) and pre-B ALL with t(1;19) (PTKi: BTK and SRC inhibitors)^[30-53]^ [Table 1]. Notably, Ph^+^ B-ALL was associated with a very poor outcome in both children and adults, with less than 20% long-term survival until the introduction of PTKi capable of inhibiting the oncogenic BCR-ABL tyrosine kinase^[36-48]^ [Table 1]. Due to very high rates of deep complete remissions and markedly improved long-term EFS and OS achieved with combinations of PTKi and standard chemo- or biotherapy, patients with Ph^+^ B-ALL are no longer considered to have a poor prognosis. The standard of care is being optimized by developing novel combinations of PTKi such as dasatinib and ponatinib with bispecific antibodies such as blinatumomab, CAR-T cells, and antibody-drug conjugates and using less toxic chemotherapy regimens^[36-48]^. Likewise, FLT3 inhibitors have contributed to improved outcomes in AML patients with a *FLT3* mutation^[49]^. FLT3 is a receptor tyrosine kinase that plays an important role in normal lymphohematopoiesis^[50]^. Leukemic cells from AML patients abundantly express FLT3 and many AML patients have activating mutations of FLT3, including internal tandem duplication mutations (FLT3-ITD) and kinase domain activation loop mutations (FLT3-ALM)^[50,51]^. *FLT3* mutations are rare in ALL, but a high-level expression of FLT3 was also reported in MLL-AF4 positive ALL patients and showed poor prognostic value^[52,53]^. Our study significantly expands the knowledge regarding FLT3 expression in ALL as well as AML by demonstrating amplified expression levels in infants, children, and adults with KMT2A/MLL-R^+^ B-ALL and children with KMT2A/MLL-R^+^ AML. Several PTKi have been approved for the treatment of AML with *FLT3* mutations, including midostaurin and gilteritinib [Table 1]. Our results suggest that FLT3 inhibitors may have clinical potential as therapeutic agents against KMT2A/MLL-R^+^ ALL. It is noteworthy that Dovitinib, a multi-functional PTKI with FLT3 inhibitory activity, was reported to exhibit nanomolar in vitro activity against KMT2A/MLL-R^+^ ALL cells^[54]^. 

**Table 1:** FDA-approved Inhibitors of FLT3, ABL1, SRC, BTK/TEC, KIT, SYK, and JAK1-3

**Target kinase**	**Drug**	**Brand**
ABL1, SRC	Bosutinib	Bosulif, SKI-606
ABL1, SRC, FGR, CKIT	Dasatinib	BMS- 354825, Sprycell
ABL1, CKIT, PDGFR	Imatinib	STI571, Gleevec
ABL1	Nilotinib	AMN107, Tasigna
ABL1	Olverembatinib	HQP1351
ABL1, SRC, FGR	Ponatinib	Iclusig
BTK/TEC	Ibrutinib	PCI-32765, Imbruvica
BTK	Zanubrutinib	BGB3111, Brukinsa
BTK	Acalabrutinib	Calquence
FLT3	Midostaurin	CPG 41251, Rydapt
FLT3	Gilteritinib	ASP2215, Xospata
JAK1	Upadacitinib	ABT-494, Rinvoq
JAK1/2	Baricitinib	Olumiant, LY 3009104
JAK1/2/3, Tyk	Ruxolitinib	Jakafi
JAK2	Fedratinib	TG101348, Inrebic
JAK3	Tofacitinib	Xeljanz
KIT/PDGFR	Ripretinib	DCC- 2618, Qinlock
SYK	Fostamatinib	R788, Tavalisse
TRKA/B/C	Larotrectinib	LOXO-101, Vitrakvi
TRKA/B/C, ROS1	Entrectinib	RXDX-101, Ignyta, Rozlytrek
VEGFR2, FLT3, CKIT	Sunitinib	Sutent

One of the intriguing findings of our study relates to the amplified expression of the gene for FLT3 in KMT2A/MLL-R^+^ pediatric AML cells. Except for GSE17855, the archived datasets used in the present study did not contain information about the FLT3 status of the respective leukemia cases. Additional KMT2A/MLL-R^+^ patients whose leukemia cells do not have amplified FLT3 gene expression but an activating *FLT3* mutation like FLT3-ITD may also benefit from the use of FLT3 inhibitors. Analysis of the GSE17855 contributed to our finding that in pediatric patients with KMT2A/MLL-R^+^ AML, the gene expression level for FLT3 is differentially amplified. It is noteworthy that only one patient with KMT2A/MLL-R^+^ AML had FLT3-ITD whereas 47 patients from the KMT2A/MLL-R negative “other” group of pediatric AML patients were FLT3-ITD^+^, including patients with cytogenetically normal AML (*N* = 18); AML with inv^[16]^ (*N* = 1); AML with t(15;17) (*N* = 12); AML with t(8;21) (*N* = 3); AML with other cytogenetic features (*N* = 9); and AML with unknown cytogenetic features (*N* = 4). Therefore, our results should not be interpreted to suggest that FLT3 inhibitors would be preferentially active in pediatric AML patients with KMT2A/MLL rearrangements, as many cases without KMT2A/MLL rearrangements whose leukemia cells are FLT3-ITD^+^ may also benefit from FLT3 inhibitors. Some PTK, such as FLT3, participate in immune suppression mediated by leukemia cells, which may promote the immune escape of leukemic clones^[55]^. Therefore, their inhibition with PTKi may partially contribute to favorable treatment outcomes by lifting the immune suppression. For example, the PTKi sorafenib has been shown to abrogate the transcriptional downregulation of interferon regulatory factor 7 (IRF7), which resulted in an augmented CD8^+^CD107a^+^IFN-γ^+^ T cell response in mouse models of FLT-ITD^+^ AML^[55]^. Mathew *et al*. proposed that sorafenib may therefore exhibit immune-mediated anti-leukemic activity in FLT3-ITD mutant AML^[55]^. 

The SRC kinase family includes several cytoplasmic PTK, including BLK, HCK, FGR, LYN, FYN, and LCK, which have important regulatory functions for signal transduction pathways related to survival, proliferation, and apoptosis of leukemic cells^[29-31]^. FDA-approved SRC kinase inhibitors have become part of the standard of care in the treatment of CML as well as Ph^+^ ALL^[36-48]^ [Table 1]. In the current study, we discovered the amplified expression of the genes for BLK, HCK, FGR as well as LYN in primary leukemic cells from infants, children as well as adults with KMT2A/MLL-R^+^ B-ALL. The gene for BLK was expressed at a 6.77-fold higher level in infant KMT2A/MLL-R^+^ ALL cells, 4.38-fold higher level in pediatric KMT2A/MLL-R^+^ ALL cells, and 21.79-fold higher level in adult KMT2A/MLL-R^+^ ALL cells than in normal hematopoietic cells. Notably, the expression of the genes for BLK, HCK and LYN in primary leukemic cells from KMT2A/MLL-R^+^ ALL patients was differentially and significantly amplified regardless of the age group compared to the expression levels in leukemic cells from B-ALL patients without KMT2A/MLL gene rearrangements. Likewise, the genes of several SRC family PTK were differentially upregulated in pediatric and adult KMT2A/MLL-R^+^ AML cells. The availability of FDA-approved potent inhibitors of SRC family PTK such as ponatinib, bosutinib, and dasatinib [Table 1] provides an opportunity to evaluate their clinical impact potential for KMT2A/MLL-R^+^ leukemias in proof-of-concept clinical trials. The insights and lessons learned in the clinical development of these PTKi as precision medicines against Ph^+^ ALL should facilitate their development as potential precision medicines against KMT2A/MLL-R^+^ ALL as well. 

Additional insights from the present study relate to the upregulation of SYK and JAK expression in pediatric and adult KMT2A/MLL-R^+^ acute leukemias. These results extend our earlier studies regarding upregulation of the JAK-STAT pathways in infant B-ALL^[56,57]^ and upregulation of SYK expression in pediatric B-ALL^[58-62]^. The availability of FDA-approved potent inhibitors of SYK and JAK provides the opportunity to evaluate their clinical efficacy in KMT2A/MLL-R^+^ acute leukemias with overexpression of their respective targets. A significant portion of high-risk acute leukemia patients, especially those with KMT2A/MLL-R^+^ ALL or AML relapse after being treated on contemporary chemotherapy protocols due to cancer drug resistance of their leukemic clones, and the survival outcome of available salvage regimens is disappointing due to the short nature and poor quality of second remissions. We previously reported that the JAK/STAT signaling pathway is constitutively active in infant pro-B ALL cells and treatment with a JAK3 inhibitor or a pan-JAK kinase inhibitor effectively triggered their apoptosis^[56,57]^. JAK targeting with a small molecule inhibitor may be a viable strategy to overcome cancer drug resistance in KMT2A/MLL-R^+^ B-ALL cells. Constitutively active JAK2-STAT5 signaling has been shown to be associated with increased surface PD-L1 expression due to amplified PD-L1 promoter activity in myeloproliferative neoplasms, which may facilitate PD-L1-mediated immune escape^[63,64]^. JAK2 inhibition reduces the PD-L1 expression levels and may therefore mitigate this risk. 

SYK has been discovered to regulate the cancer drug resistance-related anti-apoptotic STAT3, NF-κB as well as PI-3K-AKT-mTOR pathways^[60-62]^. Our earlier studies have identified high-level SYK expression as a likely contributor to cancer drug resistance and relapse in B-ALL^[60,61]^. Upregulation of SYK expression was associated with significant upregulation of at least one of the STAT3 target genes^[60]^. Inhibition of SYK caused apoptotic death in primary leukemia cells from B-ALL patients that are resistant to chemotherapy^[61]^. Notably, a nanomedicine candidate containing the SYK-inhibiting small molecule compound 1,4-bis(9-O-dihydroquinidyl) phthalazine/hydroquinidine1,4-phathalazinediyldiether (C61) was capable of destroying > 99.9% of clonogenic B-ALL cells in vivo and thereby improved the event-free survival outcome of SCID mice challenged with otherwise invariably fatal doses of human leukemic B-cell precursors in each of three different xenograft models of chemotherapy-resistant human B-ALL^[62]^. Taken together with these earlier observations, the amplified expression of SYK in the poor prognosis KMT2A/MLL-R^+^ ALL patients prompts the hypothesis that SYKi may help overcome the cancer drug resistance of relapse clones and thereby provide the foundation for more effective multi-modality treatment regimens for KMT2A/MLL-R^+^ acute leukemias.

## DECLARATIONS

### Availability of data and materials

All data are presented in the manuscript and the Supplemental Material. The publicly available archived databases that were used to generate the data have been provided in Materials and Methods. Specifically, raw Affymetrix .CEL data files on gene expression profiles of AML cells analyzed in the current study were obtained from 3 publicly available datasets deposited in the NCBI repository (GSE13159, GSE19577, and GSE17855). Raw Affymetrix .CEL data files on gene expression profiles of ALL cells analyzed in the current study were obtained from 8 publicly available datasets deposited in the NCBI repository (GSE11877, GSE13159, GSE13351, GSE18497, GSE28460, GSE7440, GSE68720, and GSE32962).

### Copyright

© The Author(s) 2022.

## References

[1] Li X, Song Y (2021). Structure, function and inhibition of critical protein-protein interactions involving mixed lineage leukemia 1 and its fusion oncoproteins. J Hematol Oncol.

[2] Arber DA, Orazi A, Hasserjian R (2016). The 2016 revision to the World Health Organization classification of myeloid neoplasms and acute leukemia. Blood.

[3] Bueno C, Montes R, Catalina P, Rodríguez R, Menendez P (2011). Insights into the cellular origin and etiology of the infant pro-B acute lymphoblastic leukemia with MLL-AF4 rearrangement. Leukemia.

[4] Britten O, Ragusa D, Tosi S, Kamel YM (2019). *MLL*-rearranged acute leukemia with t(4;11)(q21;q23)-current treatment options. is there a role for car-t cell therapy?. Cells.

[5] Kantarjian H, Kadia T, DiNardo C (2021). Acute myeloid leukemia: current progress and future directions. Blood Cancer J.

[6] Calvo C, Fenneteau O, Leverger G, Petit A, Baruchel A, Méchinaud F (2021). Infant acute myeloid leukemia: a unique clinical and biological entity. Cancers (Basel).

[7] Hoffman AE, Schoonmade LJ, Kaspers GJ (2021). Pediatric relapsed acute myeloid leukemia: a systematic review. Expert Rev Anticancer Ther.

[8] Padmakumar D, Chandraprabha VR, Gopinath P (2021). A concise review on the molecular genetics of acute myeloid leukemia. Leuk Res.

[9] Uddin R, Darwish NHE, Mousa SA (2021). Acute myeloid leukemia mutations and future mechanistic target to overcome resistance. Curr Treat Options Oncol.

[10] (2020). Chaer F, Keng M, Ballen KK. MLL-rearranged acute lymphoblastic leukemia. Curr Hematol Malig Rep.

[11] Yeung DTO, Osborn MP, White DL (2022). B-cell acute lymphoblastic leukaemia: recent discoveries in molecular pathology, their prognostic significance, and a review of the current classification. Br J Haematol.

[12] Zotova OV, Lukianova AS, Valchuk MO (2021). 11q23/MLL rearrangements in adult acute leukemia. Exp Oncol.

[13] Richard-Carpentier G, Kantarjian HM, Tang G (2021). Outcomes of acute lymphoblastic leukemia with KMT2A (MLL) rearrangement: the MD Anderson experience. Blood Adv.

[14] Goel H, Rahul E, Gupta I (2021). Molecular and genomic landscapes in secondary & therapy related acute myeloid leukemia. Am J Blood Res.

[15] Fazio G, Bardini M, De Lorenzo P (2021). Recurrent genetic fusions redefine MLL germ line acute lymphoblastic leukemia in infants. Blood.

[16] Meyer C, Burmeister T, Gröger D (2018). The MLL recombinome of acute leukemias in 2017. Leukemia.

[17] Shih LY, Liang DC, Fu JF (2006). Characterization of fusion partner genes in 114 patients with de novo acute myeloid leukemia and MLL rearrangement. Leukemia.

[18] Tomizawa D, Koh K, Sato T (2007). Outcome of risk-based therapy for infant acute lymphoblastic leukemia with or without an MLL gene rearrangement, with emphasis on late effects: a final report of two consecutive studies, MLL96 and MLL98, of the Japan Infant Leukemia Study Group. Leukemia.

[19] Reaman GH, Sposto R, Sensel MG (1999). Treatment outcome and prognostic factors for infants with acute lymphoblastic leukemia treated on two consecutive trials of the Children's Cancer Group. J Clin Oncol.

[20] Qazi S, Uckun FM (2022). Augmented expression of the IL3RA/CD123 gene in MLL/KMT2A-rearranged pediatric AML and infant ALL. Onco.

[21] Uckun FM, Qazi S (2018). Identification and targeting of CD22ΔE12 as a molecular RNAi target to overcome drug resistance in high-risk B-lineage leukemias and lymphomas. Cancer Drug Resist.

[22] Uckun FM, Myers DE, Qazi S (2015). Recombinant human CD19L-sTRAIL effectively targets B cell precursor acute lymphoblastic leukemia. J Clin Invest.

[23] Uckun FM, Mitchell LG, Qazi S (2015). Development of polypeptide-based nanoparticles for non-viral delivery of CD22 RNA Trans-splicing molecule as a new precision medicine candidate against B-lineage ALL. EBioMedicine.

[24] Uckun FM, Goodman P, Ma H, Dibirdik I, Qazi S (2010). CD22 EXON 12 deletion as a pathogenic mechanism of human B-precursor leukemia. Proc Natl Acad Sci U S A.

[25] Grembecka J, He S, Shi A (2012). Menin-MLL inhibitors reverse oncogenic activity of MLL fusion proteins in leukemia. Nat Chem Biol.

[26] Issa GC, Ravandi F, DiNardo CD, Jabbour E, Kantarjian HM, Andreeff M (2021). Therapeutic implications of menin inhibition in acute leukemias. Leukemia.

[27] Bai H, Zhang SQ, Lei H, Wang F, Ma M, Xin M (2022). Menin-MLL protein-protein interaction inhibitors: a patent review (2014-2021). Expert Opin Ther Pat.

[28] Tsakaneli A, Williams O Drug repurposing for targeting acute leukemia with. KMT2A.

[29] Roskoski R Jr (2020). The role of small molecule Flt3 receptor protein-tyrosine kinase inhibitors in the treatment of Flt3-positive acute myelogenous leukemias. Pharmacol Res.

[30] Bhullar KS, Lagarón NO, McGowan EM (2018). Kinase-targeted cancer therapies: progress, challenges and future directions. Mol Cancer.

[31] Scheijen B, Griffin JD (2002). Tyrosine kinase oncogenes in normal hematopoiesis and hematological disease. Oncogene.

[32] Ribera JM (2021). Philadelphia chromosome-like acute lymphoblastic leukemia. Still a pending matter. Haematologica.

[33] Jain N, Jabbour EJ, McKay PZ (2017). Ruxolitinib or dasatinib in combination with chemotherapy for patients with relapsed/refractory Philadelphia (Ph)-like acute lymphoblastic leukemia: a phase I-II trial. Blood.

[34] van der Veer A, van der Velden VH, Willemse ME (2014). Interference with pre-B-cell receptor signaling offers a therapeutic option for TCF3-rearranged childhood acute lymphoblastic leukemia. Blood Cancer J.

[35] Buchner M, Müschen M (2014). Targeting the B-cell receptor signaling pathway in B lymphoid malignancies. Curr Opin Hematol.

[36] Slayton WB, Schultz KR, Kairalla JA (2018). Dasatinib plus intensive chemotherapy in children, adolescents, and young adults with Philadelphia chromosome-positive acute lymphoblastic leukemia: results of Children’s Oncology Group Trial AALL0622. J Clin Oncol.

[37] Schultz KR, Carroll A, Heerema NA (2014). ; Children’s Oncology Group. Long-term follow-up of imatinib in pediatric Philadelphia chromosome-positive acute lymphoblastic leukemia: Children’s Oncology Group study AALL0031. Leukemia.

[38] Warraich Z, Tenneti P, Thai T (2020). Relapse prevention with tyrosine kinase inhibitors after allogeneic transplantation for philadelphia chromosome-positive acute lymphoblast leukemia: a systematic review. Biol Blood Marrow Transplant.

[39] Foà R, Bassan R, Vitale A, GIMEMA Investigators (2020). Dasatinib-blinatumomab for ph-positive acute lymphoblastic leukemia in adults. N Engl J Med.

[40] Foà R, Vitale A, Vignetti M, GIMEMA Acute Leukemia Working Party (2011). Dasatinib as first-line treatment for adult patients with Philadelphia chromosome-positive acute lymphoblastic leukemia. Blood.

[41] Samra B, Jabbour E, Ravandi F, Kantarjian H, Short NJ (2020). Evolving therapy of adult acute lymphoblastic leukemia: state-of-the-art treatment and future directions. J Hematol Oncol.

[42] Jabbour E, Kantarjian H, Ravandi F (2015). Combination of hyper-CVAD with ponatinib as first-line therapy for patients with Philadelphia chromosome-positive acute lymphoblastic leukaemia: a single-centre, phase 2 study. Lancet Oncol.

[43] Short NJ, Kantarjian HM, Ravandi F (2019). Long-term safety and efficacy of hyper-CVAD plus ponatinib as frontline therapy for adults with Philadelphia chromosome-positive acute lymphoblastic leukemia. Blood.

[44] Rousselot P, Coude MM, Gokbuget N (2016). Dasatinib and low-intensity chemotherapy in elderly patients with Philadelphia chromosome-positive ALL. Blood.

[45] Chiaretti S, Bassan R, Vitale A (2019). Dasatinib-blinatumomab combination for the front-line treatment of adult Ph + ALL patients. Updated results of the Gimema LAL2116 D-Alba trial. Blood.

[46] Jabbour E, DerSarkissian M, Duh MS (2018). Efficacy of ponatinib versus earlier generation tyrosine kinase inhibitors for front-line treatment of newly diagnosed Philadelphia-positive acute lymphoblastic leukemia. Clin Lymphoma Myeloma Leuk.

[47] Jain N, Cortes JE, Ravandi F (2017). Inotuzumab ozogamicin in combination with bosutinib for patients with relapsed or refractory Ph + ALL or CML in lymphoid blast phase. Blood.

[48] Chen M, Zhu Y, Lin Y, Tengwang T, Zhang L (2021). Use of tyrosine kinase inhibitors for paediatric Philadelphia chromosome-positive acute lymphoblastic leukaemia: a systematic review and meta-analysis. BMJ Open.

[49] Short NJ, Kantarjian H, Jabbour E (2022). SOHO state of the art updates & next questions: intensive and non-intensive approaches for adults with Philadelphia chromosome-positive acute lymphoblastic leukemia. Clin Lymphoma Myeloma Leuk.

[50] Gilliland DG, Griffin JD (2002). The roles of FLT3 in hematopoiesis and leukemia. Blood.

[51] Meshinchi S, Alonzo TA, Stirewalt DL (2006). Clinical implications of FLT3 mutations in pediatric AML. Blood.

[52] Chillón MC, Gómez-Casares MT, López-Jorge CE (2012). Prognostic significance of FLT3 mutational status and expression levels in MLL-AF4+ and MLL-germline acute lymphoblastic leukemia. Leukemia.

[53] Zhang Y, Zhang Y, Wang F (2020). The mutational spectrum of FLT3 gene in acute lymphoblastic leukemia is different from acute myeloid leukemia. Cancer Gene Ther.

[54] Eucker J, Zang C, Zhou Y TKI258, a multi-tyrosine kinase inhibitor is efficacious against human infant/childhood lymphoblastic leukemia in vitro. Anticancer Res.

[55] Mathew NR, Baumgartner F, Braun L (2018). Sorafenib promotes graft-versus-leukemia activity in mice and humans through IL-15 production in FLT3-ITD-mutant leukemia cells. Nat Med.

[56] Qazi S, Uckun FM (2010). Gene expression profiles of infant acute lymphoblastic leukaemia and its prognostically distinct subsets. Br J Haematol.

[57] Uckun FM, Pitt J, Qazi S (2011). JAK3 pathway is constitutively active in B-lineage acute lymphoblastic leukemia. Expert Rev Anticancer Ther.

[58] Uckun FM, Qazi S (2014). SYK as a new therapeutic target in B-cell precursor acute lymphoblastic leukemia. J Cancer Ther.

[59] Uckun FM, Qazi S (2010). Spleen tyrosine kinase as a molecular target for treatment of leukemias and lymphomas. Exp Rev Antic Ther.

[60] Uckun FM, Qazi S, Ma H, Tuel-Ahlgren L, Ozer Z (2010). STAT3 is a substrate of syk tyrosine kinase in b-lineage leukemia/lymphoma cells exposed to oxidative stress. Proc Natl Acad Sci U S A.

[61] Uckun FM, Ek RO, Jan ST, Chen CL, Qazi S (2010). Targeting syk kinase-dependent anti-apoptotic resistance pathway in b-lineage acute lymphoblastic leukemia (all) cells with a potent SYK inhibitory pentapeptide mimic. Brit J Hematol.

[62] Uckun FM, Qazi S, Cely I (2013). Nanoscale liposomal formulation of a SYK p-site inhibitor against b-precursor leukemia. Blood.

[63] Milosevic Feenstra JD, Jäger R, Schischlik F (2022). PD-L1 overexpression correlates with JAK2-V617F mutational burden and is associated with 9p uniparental disomy in myeloproliferative neoplasms. Am J Hematol.

[64] Prestipino A, Emhardt AJ, Aumann K (2018). Oncogenic JAK2^V617F^ causes PD-L1 expression, mediating immune escape in myeloproliferative neoplasms. Sci Transl Med.

