# Supplementary material for: Tyrosine kinases in KMT2A/MLL-rearranged acute leukemias as potential therapeutic targets to overcome cancer drug resistance
Source: Cancer Drug Resist. 2022 Oct 9;5(4):902–16. doi: 10.20517/cdr.2022.78 (PMC9771742; doi:10.20517/cdr.2022.78)
Supplement: Supplementary file 1 [file cdr-5-4-902-SupplementaryMaterials.pdf]

Supplemental Materials

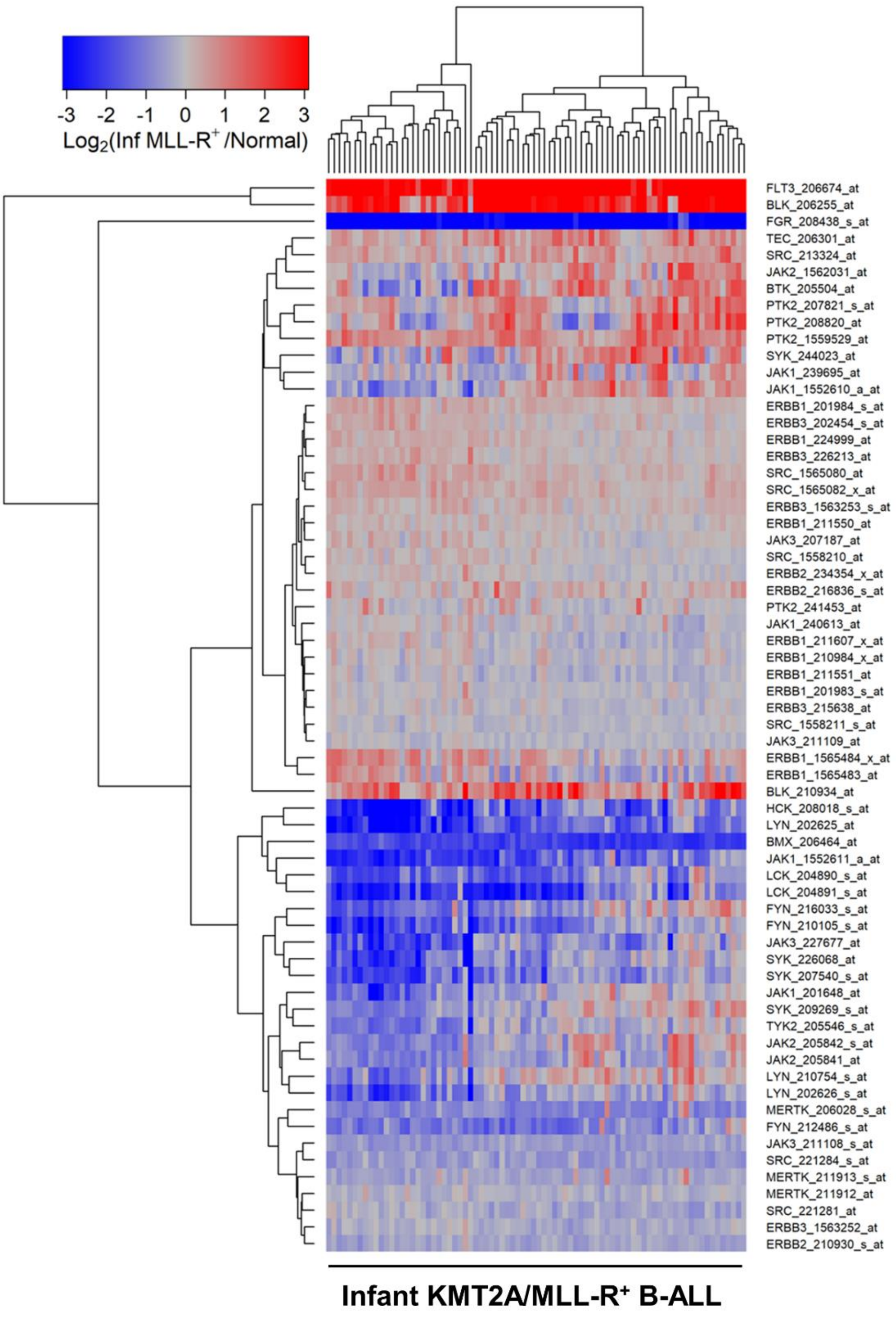

Figure S1. Gene Expression Levels for Tyrosine Kinases in Leukemic Cells from Infants with KMT2A/MLL-R$^+$ B-ALL vs. Normal Hematopoietic Cells. We examined the gene expression data in the archived data infant ALL dataset GSE68720 and the control dataset from GSE13159. Infant KMT2A/MLL-R$^+$ B-ALL gene partners for KMT2A were AF4 (N=48), ENL (N=16), AF9 (N=6), ASAH3 (N=1), EPS15 (N=3), Unknown (N=6) (GSE68720; Total N = 80). The cluster figure displays the expression levels in KMT2A/MLL-R$^+$ ALL cells mean centered to the reference group (normal bone marrow samples) for log_2-transformed fold change values (blue represents under expression and red color represents over expression in KMT2A/MLL-R$^+$ samples). The expression levels of co-regulated probesets for both probesets (rows) and patients (columns) are organized in the depicted dendrograms. The comparison of the log2 transformed RMA values for normal hematopoietic cells from 74 control samples with RMA values for leukemic blasts from 80 infants with KMT2A/MLL-R$^+$ ALL showed 43 significantly dysregulated probesets of which 17 were upregulated in KMT2A/MLL-R$^+$ cases (cluster figure depicts under and overexpressed probesets represented by blue to red color respectively). FLT3_206674_at was the most significantly upregulated transcript in MLL-R$^+$ subset of cases (Fold Change = 16.09; P-value < 10^{-8}) followed by BLK_206255_at (Fold Change = 6.77; P-value < 10^{-8}) and BLK_210934_at (Fold Change = 2.43; P-value < 10^{-8}) (**Table S1**) that also formed a co-regulated cluster of expression changes. TEC,

SRC, JAK2, BTK, PTK2 (3 probesets), SYK and JAK1 (2 probesets) were also observed to form a cluster of upregulated genes.

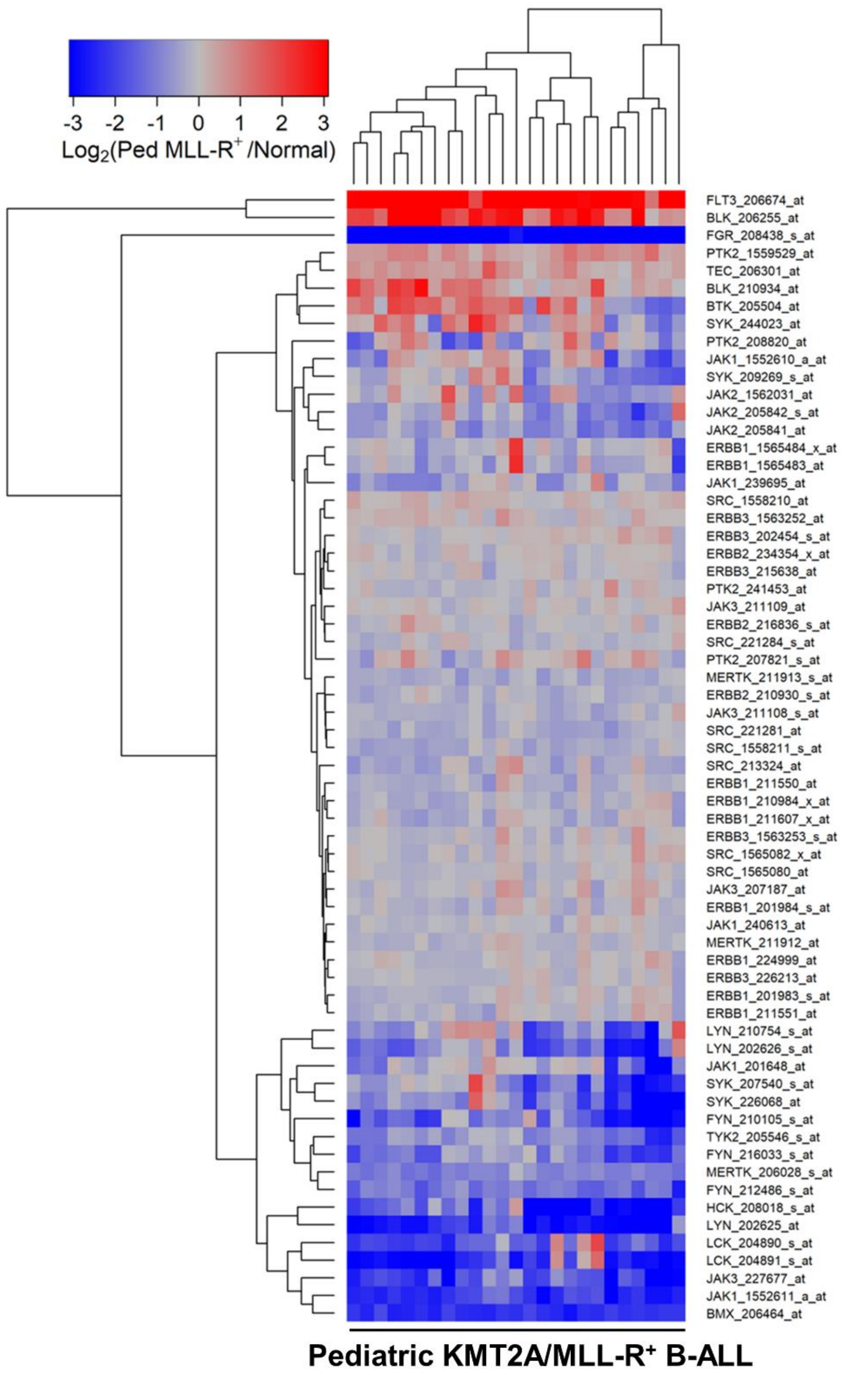

Figure S2. Gene Expression Levels for Tyrosine Kinases in Leukemic Cells from Pediatric Patients with KMT2A/MLL-R+ B-ALL vs. Normal Hematopoietic Cells. We examined the gene expression data in the archived pediatric ALL data sets GSE11877 and GSE13351 and the control dataset from GSE13159. The cluster figure displays the expression levels in KMT2A/MLL-R+ ALL cells mean centered to the reference group (normal bone marrow samples) for log_2-transformed fold change values (blue represents under expression and red color represents over expression in KMT2A/MLL-R+ samples). The expression levels of co-regulated probesets for both probesets (rows) and patients (columns) are organized in the depicted dendrograms. Depicted are the differential gene expression changes of log_2-transformed, robust multi-array analysis (RMA) normalized values for 25 pediatric patients with KMT2A/MLL-R+ ALL (GSE11877 and GSE13351). The expression levels in KMT2A/MLL-R+ ALL cells were mean centered to the mean expression of 74 normal control samples (GSE13159) and visualized using a two-way clustering algorithm to determine co-regulation of Tyrosine Kinase genes. This analysis exhibited 32 dysregulated probesets of which 6 were significantly upregulated in KMT2A/MLL-R+ subset of cases. FLT3_206674_at was the most significantly upregulated transcript in KMT2A/MLL-R+ subset of cases (Fold Change = 22.38; P-value < 10^{-8}) followed by

BLK_206255_at (Fold Change = 4.38; P-value < 10^{-8}) and BLK_210934_at (Fold Change = 1.79; P-value < 10^{-8}). (**Table S2**).

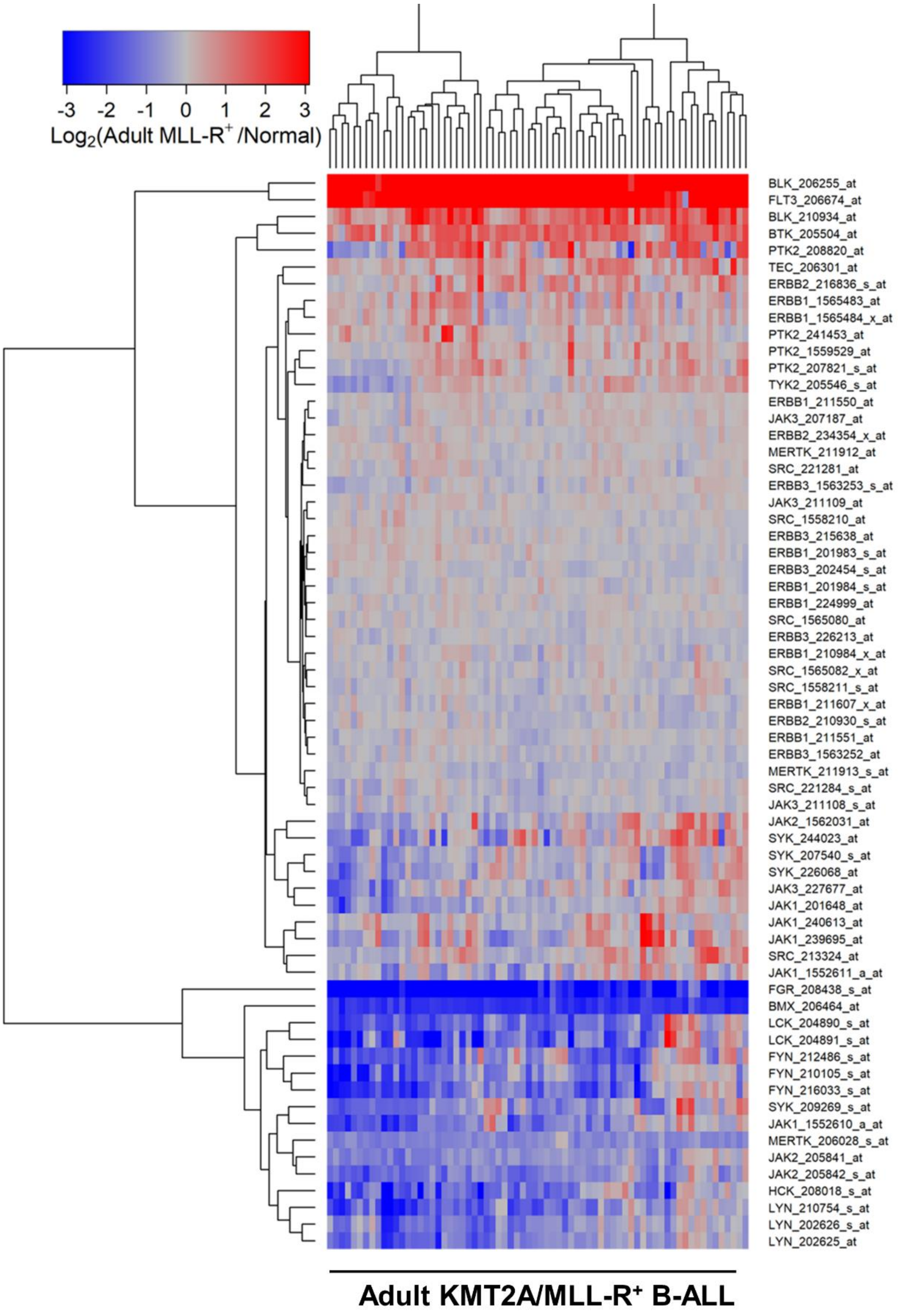

Figure S3. Gene Expression Levels for Tyrosine Kinases in Leukemic Cells from Adult Patients with KMT2A/MLL-R$^+$ B- ALL vs. Normal Hematopoietic Cells. We examined the gene expression data in the archived data set GSE13159. The cluster figure displays the expression levels in KMT2A/MLL-R$^+$ ALL cells mean centered to the reference group (normal bone marrow samples) for log2 transformed fold change values (blue represents under expression and red color represents over expression in KMT2A/MLL-R$^+$ samples). The expression levels of co-regulated probesets for both probesets (rows) and patients (columns) are organized in the depicted dendrograms. The comparison of the log2 transformed RMA values for normal hematopoietic cells from 74 control samples with RMA values for leukemic blasts from 70 adult patients with KMT2A/MLL-R$^+$ ALL showed 31 dysregulated probesets of which 13 were upregulated in adult KMT2A/MLL-R$^+$ subset of cases. BLK_206255_at was the most significantly upregulated probeset (Fold Change = 21.79; P-value < 10^{-8}) followed by FLT3_206674_at (Fold Change = 21; P-value < 10^{-8}) and

BLK_210934_at (Fold Change = 2.88; P-value < 10^{-8}). (**Table S3**) which formed a co-regulated cluster of expression changes.

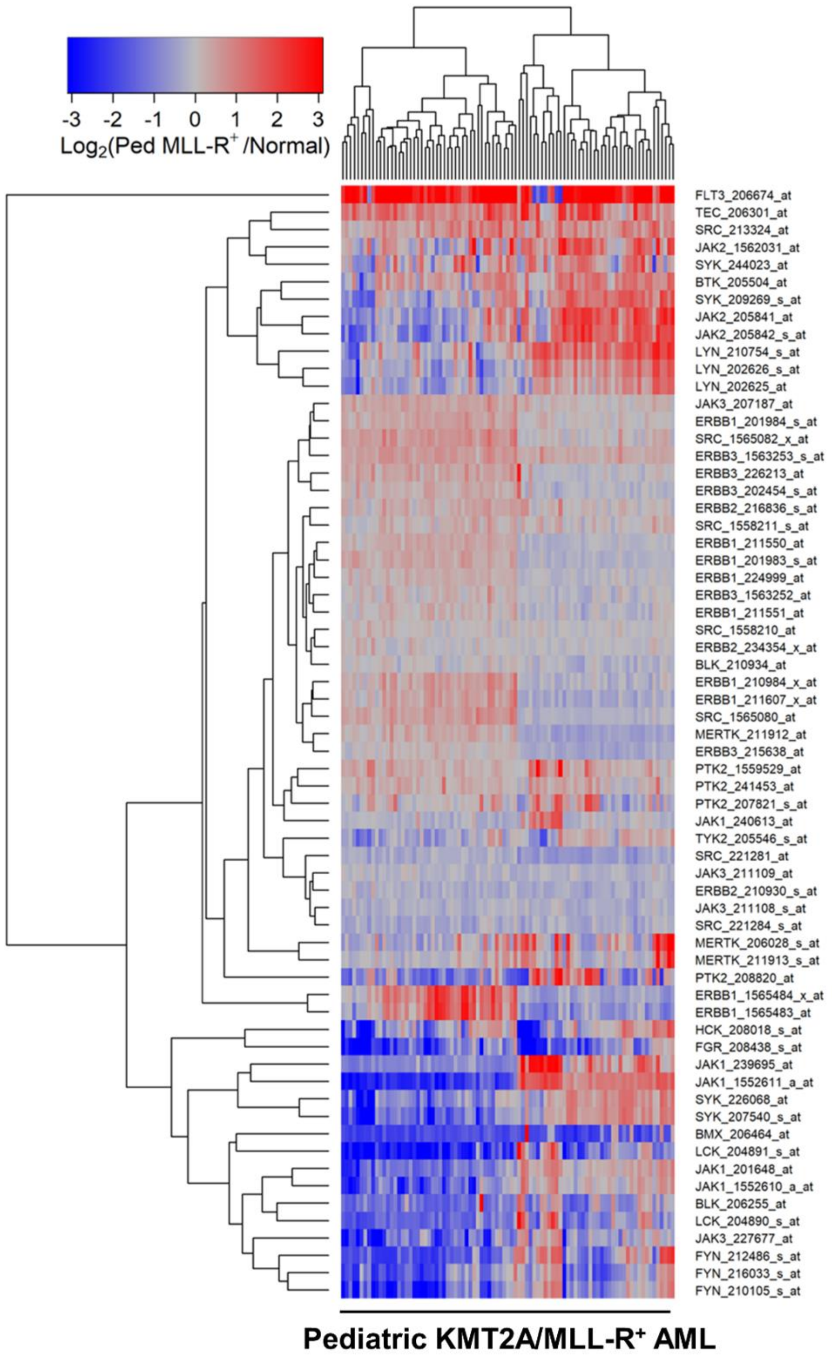

Figure S4. Gene Expression Levels for Tyrosine Kinases in Leukemic Cells from Pediatric Patients with KMT2A/MLL-R⁺ AML vs. Normal Hematopoietic Cells. We examined the gene expression data in the archived data sets GSE13159, GSE17855 and GSE19577.The cluster figure displays the expression levels in KMT2A/MLL-R⁺ AML cells mean centered to the reference group (normal bone marrow samples) for log_2-transformed fold change values (blue represents under expression and red color represents over expression in KMT2A/MLL-R⁺ samples). The expression levels of co-regulated probesets for both probesets (rows) and patients (columns) are organized in the depicted dendrograms. Depicted are the differential gene expression changes of log_2-transformed, robust multi-array analysis (RMA) normalized values for 89 pediatric patients with KMT2A/MLL-R⁺ AML (GSE17855 (N=47) and GSE19577(N=42)). The expression levels in KMT2A/MLL-R⁺ AML cells were mean centered to the mean expression of 74 normal control samples (GSE13159) and visualized using a two-way clustering algorithm to determine co-regulation of Tyrosine Kinase genes (red color for overexpression). This analysis exhibited 38 differentially regulated probesets of which 19 were upregulated in pediatric AML with KMT2A/MLL-R⁺ subset of cases. FLT3_206674_at was the most significantly upregulated probeset (Fold Change = 7.54; P-value < 10^{-8}) followed by TEC_206301_at (Fold Change = 2.2; P-value < 10^{-8}) and SRC_213324_at (Fold Change = 1.64; P-value < 10^{-8}) (**Table S7**).

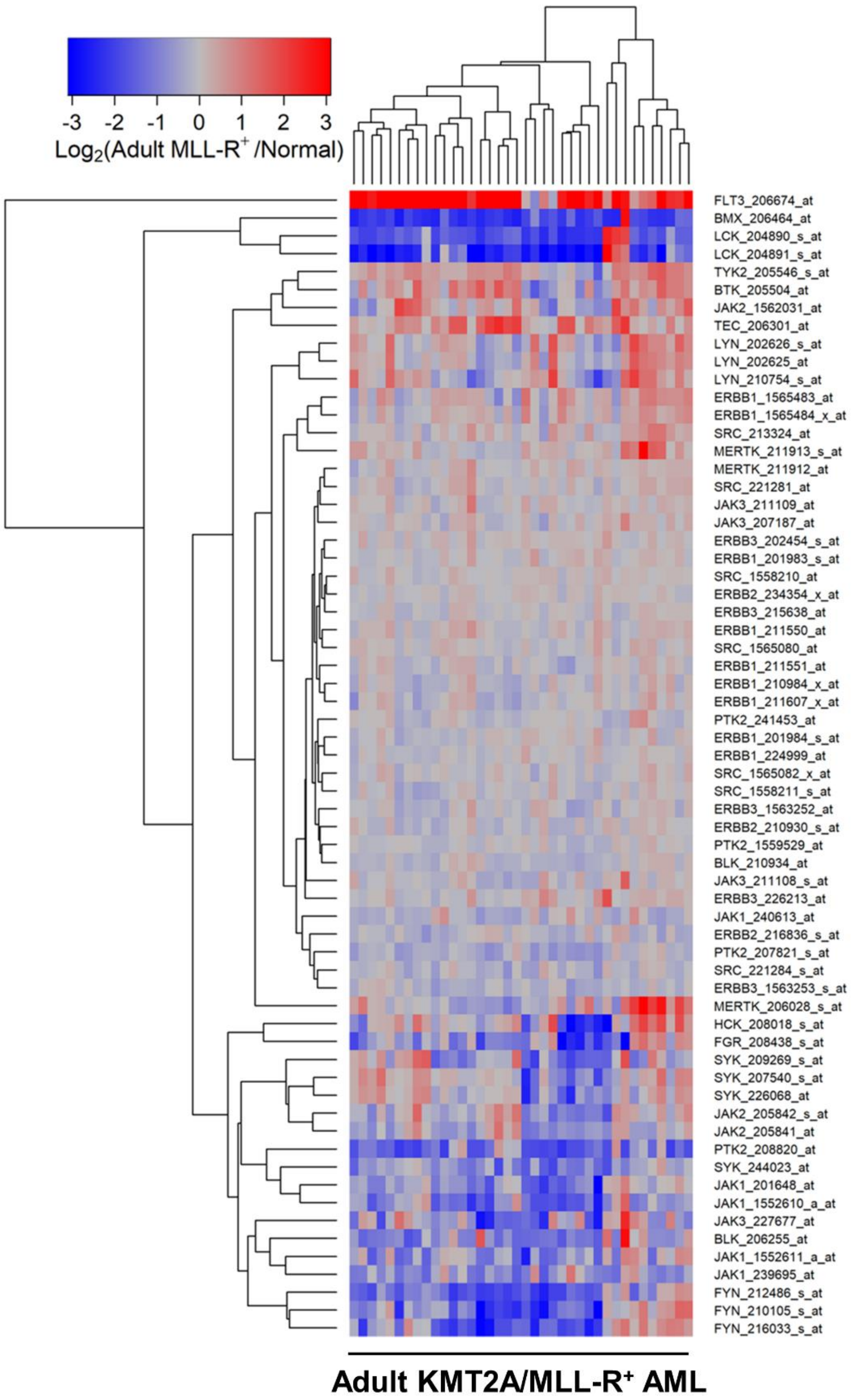

Figure S5. Gene Expression Levels for Tyrosine Kinases in Leukemic Cells from Adult Patients with KMT2A/MLL-R$^+$ AML vs. Normal Hematopoietic Cells. We examined the gene expression data in the archived data set GSE13159. The cluster figure displays the expression levels in KMT2A/MLL-R$^+$ AML cells mean centered to the reference group (normal bone marrow samples) for log_2-transformed fold change values (blue represents under expression and red color represents over expression in KMT2A/MLL-R$^+$ samples). The expression levels of co-regulated probesets for both probesets (rows) and patients (columns) are organized in the depicted dendrograms. Comparison of 74 cases of normal bone marrow samples with 38 cases of Adult AML with KMT2A/MLL-R$^+$ revealed 23 probesets that were dysregulated, of which 6 were upregulated in Adult AML subset of cases. FLT3_206674_at was the most significantly upregulated probeset (Fold Change = 8.28; P-value < 10^{-8}) followed by TEC_206301_at (Fold Change = 1.73; P-value < 10^{-8}) and TYK2_205546_s_at (Fold Change = 1.39; P-value = 8.5 x 10^{-5}).(**Table S8**).

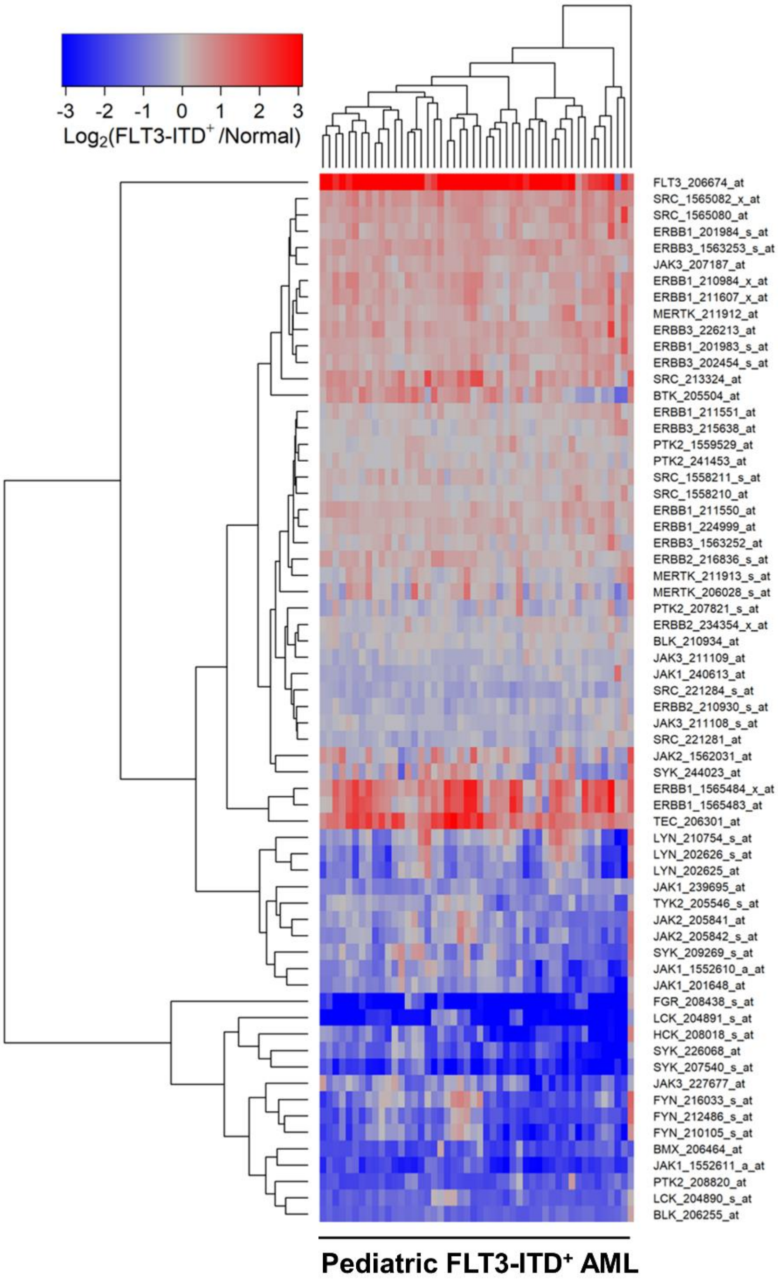

Figure S6. Gene Expression Levels for Tyrosine Kinases in Leukemic Cells from Pediatric AML Patients with FLT3-ITD$^+$ mutation vs Normal Hematopoietic Cells.

We examined the gene expression data in the archived data sets GSE17855 and GSE13159. The cluster figure displays the expression levels in FLT3-ITD$^+$ AML cells mean centered to the reference group (Normal Hematopoietic Cells) for log2 transformed fold change values (blue represents under expression and red color represents over expression in FLT3-ITD$^+$ samples). The expression levels of co-regulated probesets for both probesets (rows) and patients (columns) are organized in the depicted dendrograms. Comparing 74 cases of normal samples (GSE13159) with 48 cases of FLT3-ITD$^+$ pediatric AML samples (GSE17855) exhibited 48 differentially expressed probesets of which 20 probesets were significantly upregulated in pediatric FLT3-ITD$^+$ subset of cases. FLT3_206674_at was the most significantly upregulated probeset in FLT3-ITD$^+$ subset of cases (Fold Change = 9.01; P-value < 10^{-8}) followed by TEC_206301_at (Fold Change = 2.58; P-value < 10^{-8}) and ERBB1_1565484_x_at (Fold Change = 2.24; P-value < 10^{-8}). (**Table S11**)

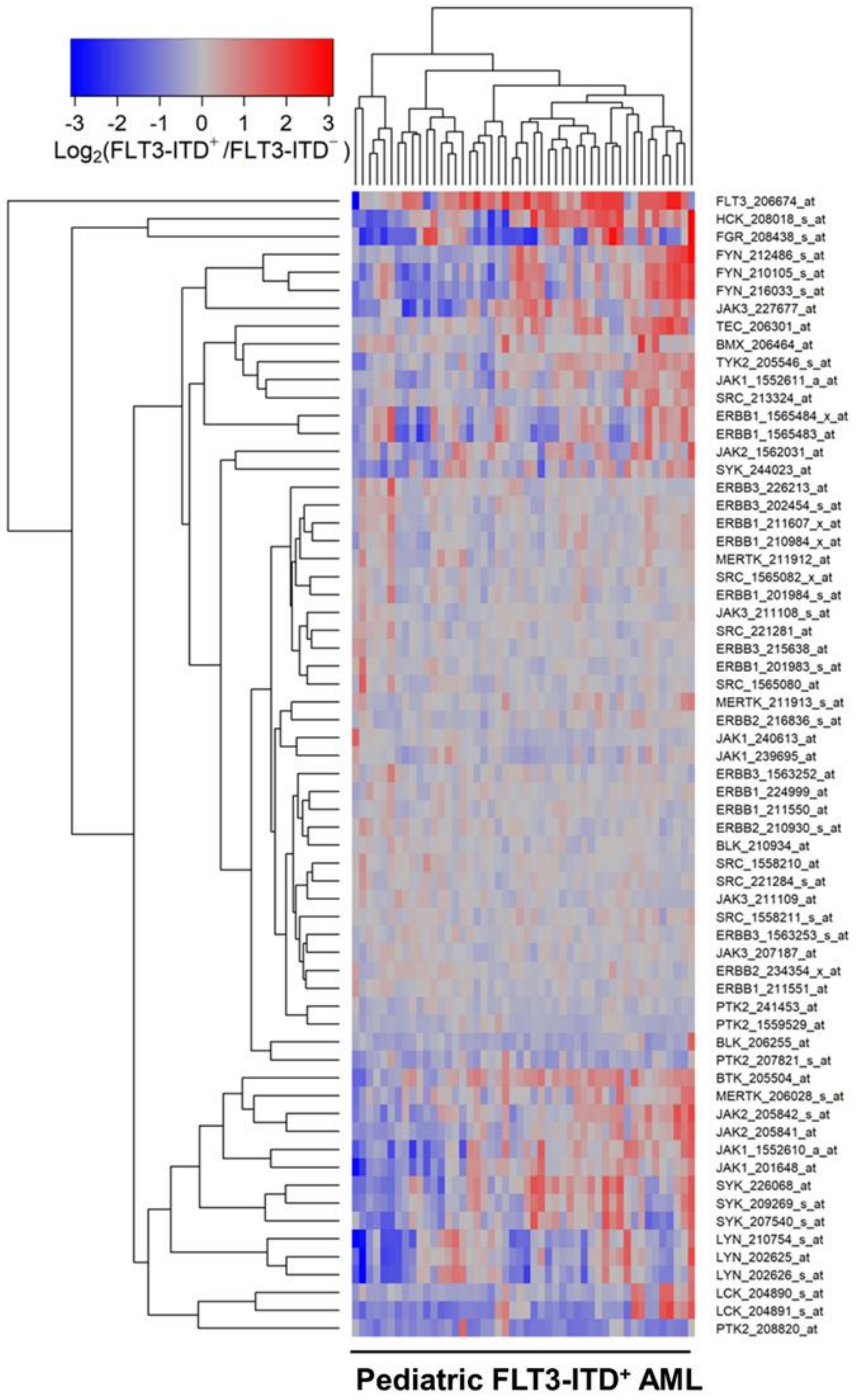

Figure S7. Gene Expression Levels for Tyrosine Kinases in Leukemic Cells from Pediatric AML Patients with FLT3-ITD^{+} mutation vs FLT3-ITD^{-}.

We examined the gene expression data in the archived data sets GSE17855. The cluster figure displays the expression levels in FLT3-ITD^{+} AML cells mean centered to the reference group (FLT3-ITD^{-}) for log2 transformed fold change values (blue represents under expression and red color represents over expression in FLT3-ITD^{+} samples). The expression levels of co-regulated probesets for both probesets (rows) and patients (columns) are organized in the depicted dendrograms. Comparing 189 cases of FLT3-ITD^{-} samples (GSE17855) with 48 cases of FLT3-ITD^{+} pediatric AML samples (GSE17855) exhibited 13 differentially expressed probesets of which 4 probesets were significantly upregulated in pediatric FLT3-ITD^{+} subset of cases. FLT3_206674_at was the most significantly upregulated probeset (Fold Change = 1.96; P-value < 10^{-8}) followed by BTK_205504_at (Fold Change = 1.25; P-value = 9.8×10^{-4}) and TEC_206301_at (Fold Change = 1.23; P-value = 0.0025). (**Table S12**).

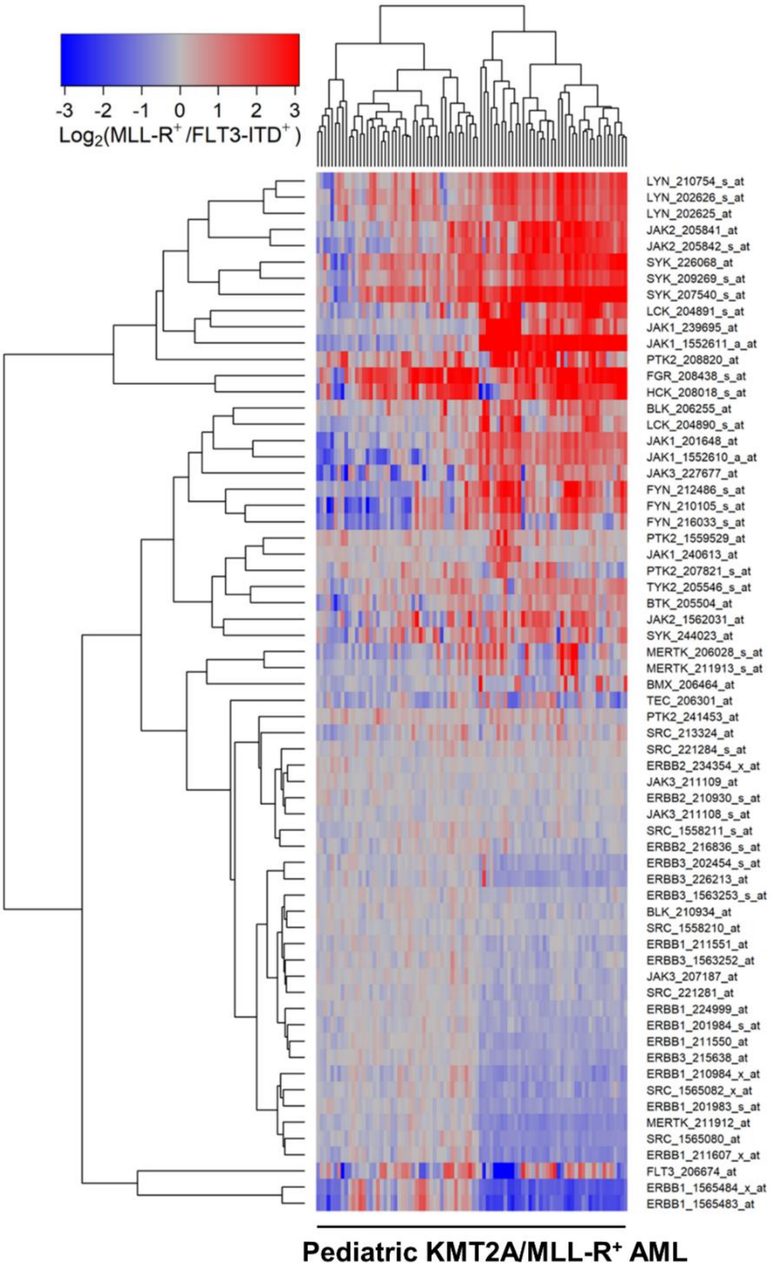

Figure S8. Gene Expression Levels for Tyrosine Kinases in Leukemic Cells from vs. Pediatric AML Patients with KMT2A/MLL-R$^+$ vs. Pediatric AML Patients with FLT3-ITD$^+$ mutation.

We examined the gene expression data in the archived data sets GSE19577 and GSE17855. The cluster figure displays the expression levels in KMT2A/MLL-R$^+$ AML cells mean centered to the reference group (Patients with FLT3-ITD$^+$) for log2 transformed fold change values (blue represents under expression and red color represents over expression in KMT2A/MLL-R$^+$ samples). The expression levels of co-regulated probesets for both probesets (rows) and patients (columns) are organized in the depicted dendrograms. Comparing 88 cases of KMT2A/MLL-R$^+$ (GSE17855 (N=46); GSE19577 (N=42)) with 47 cases of FLT3-ITD$^+$ pediatric AML samples (GSE17855) exhibited 31 differentially expressed probesets of which 24 probesets were significantly upregulated in pediatric KMT2A/MLL-R$^+$ cases. FGR_208438_s_at was the most significantly upregulated transcript in KMT2A/MLL-R$^+$ cases (Fold Change = 4.40; P-value < 10^{-8}) followed by SYK_207540_s_at (Fold Change = 3.81; P-value < 10^{-8}) and JAK1_1552611_a_at (Fold Change = 2.78; P-value < 10^{-8}). ERBB1_1565483_at was the most significantly downregulated transcript in KMT2A/MLL-R$^+$ cases (Fold Change = 0.56; P-value < 10^{-8}) followed by ERBB1_1565484_x_at (Fold Change = 0.57; Pval = 2.5 x 10^{-8}) and MERTK_211912_at (Fold Change = 0.73; Pval = 0.0016). (**Table S13**)

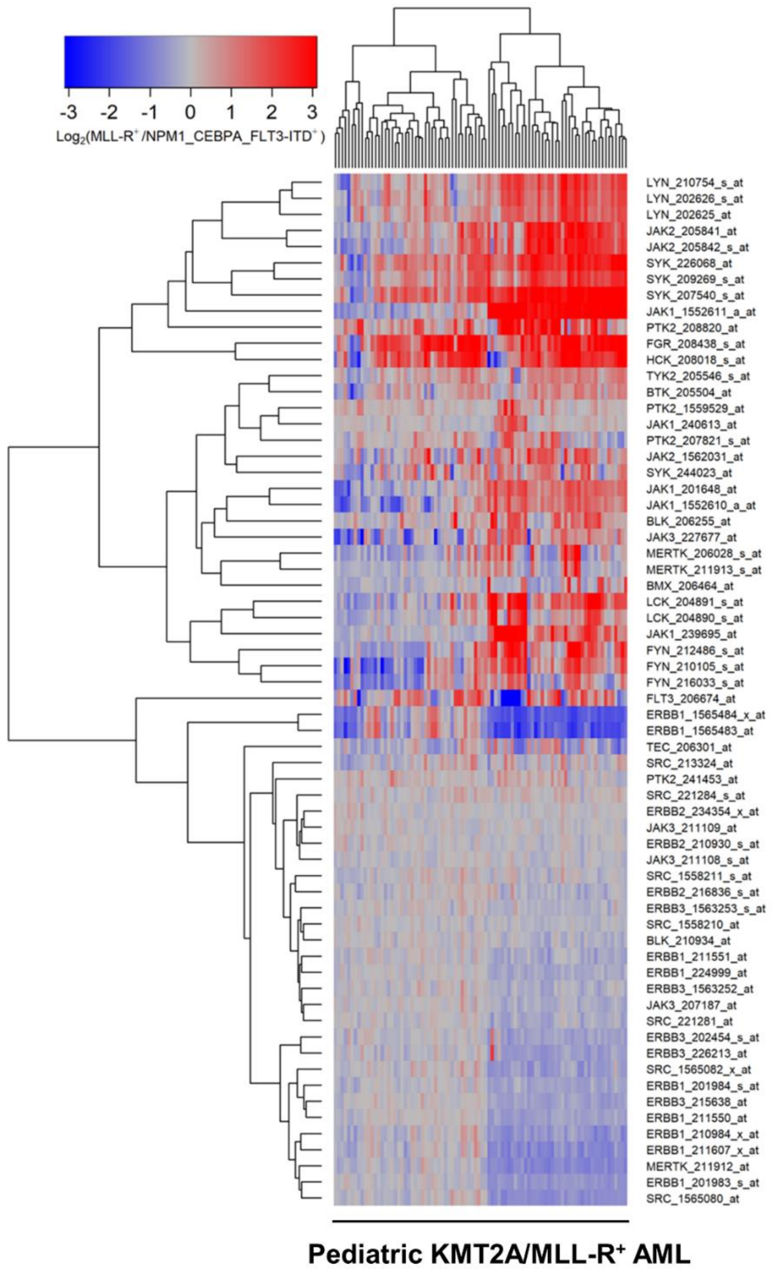

Figure S9. Gene Expression Levels for Tyrosine Kinases in Leukemic Cells from Pediatric Patients with KMT2A/MLL-R⁺ AML vs. Pediatric AML patients with FLT3-ITD⁺ , NPM1⁺ or CEBPA⁺.

We examined the gene expression data in the archived data sets GSE19577 and GSE17855. The cluster figure displays the expression levels in KMT2A/MLL-R$^+$ AML cells mean centered to the reference group (Pediatric AML patients with FLT3-ITD$^+$, NPM1$^+$ or CEBPA$^+$.) for log_2-transformed fold change values (blue represents under expression and red color represents over expression in KMT2A/MLL-R$^+$ samples). The expression levels of co-regulated probesets for both probesets (rows) and patients (columns) are organized in the depicted dendrograms. One case that harbored both FLT3-ITD$^+$ and KMT2A/MLL-R$^+$ mutations was removed from the comparison. Comparing 88 cases of KMT2A/MLL-R$^+$ (GSE17855 (N=46); GSE19577 (N=42)) with 71 cases of FLT3-ITD$^+$ /NPM1$^+$/CEBPA$^+$ pediatric AML samples exhibited 34 differentially expressed probesets of which 26 probesets were significantly upregulated in pediatric MLL-R$^+$ subset of cases. FGR_208438_s_at was the most significantly upregulated probeset in KMT2A/MLL-R$^+$ cases (Fold Change = 4.31; P-value $< 10^{-8}$) followed by SYK_207540_s_at (Fold Change = 3.92; P-value $< 10^{-8}$) and HCK_208018_s_at (Fold Change = 2.86; P-value $< 10^{-8}$). ERBB1_1565483_at was the most significantly downregulated transcript in KMT2A/MLL-R$^+$ cases (Fold Change = 0.55; P-value $< 10^{-8}$) followed by ERBB1_1565484_x_at (Fold Change = 0.56; P-value $< 10^{-8}$) and MERTK_211912_at (Fold Change = 0.72; P-value = 1.5×10^{-4}). (**Table S14**).

Table S1. Gene Expression Levels for Tyrosine Kinases in Leukemic Cells from Infants with KMT2A/MLL-R$^+$ B-ALL vs. Normal Hematopoietic Cells.

Probeset	Fold Change (Infant ALL MLL-R$^+$ /Normal)	Linear Contrast (P-value)
FLT3_206674_at	16.09	<1 x 10 $^{-8}$
BLK_206255_at	6.77	<1 x 10 $^{-8}$
BLK_210934_at	2.43	<1 x 10 $^{-8}$
PTK2_1559529_at	1.84	<1 x 10 $^{-8}$
TEC_206301_at	1.56	<1 x 10 $^{-8}$
PTK2_207821_s_at	1.46	3.0 x 10 $^{-7}$
SRC_213324_at	1.41	4.8 x 10 $^{-6}$
PTK2_208820_at	1.39	8.2 x 10 $^{-6}$
BTK_205504_at	1.36	3.8 x 10 $^{-5}$
ERBB1_1565484_x_at	1.34	8.8 x 10 $^{-5}$
SYK_244023_at	1.31	2.9 x 10 $^{-4}$
SRC_1565080_at	1.28	1.1 x 10 $^{-3}$
JAK2_1562031_at	1.27	1.2 x 10 $^{-3}$
SRC_1565082_x_at	1.26	2.0 x 10 $^{-3}$
ERBB1_201984_s_at	1.23	6.3 x 10 $^{-3}$
ERBB2_216836_s_at	1.19	2.1 x 10 $^{-2}$
ERBB3_1563253_s_at	1.17	3.8 x 10 $^{-2}$
ERBB1_224999_at	1.15	5.5 x 10 $^{-2}$
ERBB3_202454_s_at	1.15	5.8 x 10 $^{-2}$
ERBB3_226213_at	1.14	8.4 x 10 $^{-2}$
JAK1_239695_at	1.08	2.9 x 10 $^{-1}$
ERBB1_211550_at	1.08	3.0 x 10 $^{-1}$
JAK3_207187_at	1.08	3.1 x 10 $^{-1}$
JAK1_1552610_a_at	1.07	3.5 x 10 $^{-1}$
ERBB1_1565483_at	1.06	4.5 x 10 $^{-1}$
PTK2_241453_at	1.04	6.0 x 10 $^{-1}$
SRC_1558210_at	1.03	6.6 x 10 $^{-1}$
ERBB2_234354_x_at	1.02	7.5 x 10 $^{-1}$
ERBB1_210984_x_at	0.99	9.0 x 10 $^{-1}$
JAK1_240613_at	0.98	7.9 x 10 $^{-1}$
ERBB1_201983_s_at	0.98	7.6 x 10 $^{-1}$
ERBB3_215638_at	0.96	5.5 x 10 $^{-1}$
ERBB1_211607_x_at	0.95	5.0 x 10 $^{-1}$
SRC_1558211_s_at	0.94	3.7 x 10 $^{-1}$
JAK3_211109_at	0.93	2.9 x 10 $^{-1}$
ERBB1_211551_at	0.9	1.5 x 10 $^{-1}$
LYN_210754_s_at	0.89	1.3 x 10 $^{-1}$
MERTK_211912_at	0.87	6.4 x 10 $^{-2}$
SYK_209269_s_at	0.86	3.6 x 10 $^{-2}$
JAK2_205842_s_at	0.84	1.6 x 10 $^{-2}$
ERBB3_1563252_at	0.83	1.3 x 10 $^{-2}$
SRC_221281_at	0.79	1.5 x 10 $^{-3}$
MERTK_211913_s_at	0.78	7.9 x 10 $^{-4}$
JAK2_205841_at	0.77	4.4 x 10 $^{-4}$
ERBB2_210930_s_at	0.76	1.5 x 10 $^{-4}$
TYK2_205546_s_at	0.69	4.0 x 10 $^{-7}$
JAK3_211108_s_at	0.68	2.0 x 10 $^{-7}$
JAK1_201648_at	0.67	1.0 x 10 $^{-7}$
SRC_221284_s_at	0.67	1.0 x 10 $^{-7}$
FYN_216033_s_at	0.67	<1 x 10 $^{-8}$
LYN_202626_s_at	0.56	<1 x 10 $^{-8}$
MERTK_206028_s_at	0.55	<1 x 10 $^{-8}$
SYK_226068_at	0.52	<1 x 10 $^{-8}$
FYN_212486_s_at	0.51	<1 x 10 $^{-8}$
JAK3_227677_at	0.47	<1 x 10 $^{-8}$
SYK_207540_s_at	0.45	<1 x 10 $^{-8}$
LCK_204890_s_at	0.45	<1 x 10 $^{-8}$
FYN_210105_s_at	0.42	<1 x 10 $^{-8}$
JAK1_1552611_a_at	0.32	<1 x 10 $^{-8}$
HCK_208018_s_at	0.3	<1 x 10 $^{-8}$
LCK_204891_s_at	0.28	<1 x 10 $^{-8}$
LYN_202625_at	0.27	<1 x 10 $^{-8}$
BMX_206464_at	0.24	<1 x 10 $^{-8}$
FGR_208438_s_at	0.04	<1 x 10 $^{-8}$

Table S2. Gene Expression Levels for Tyrosine Kinases in Leukemic Cells from Pediatric Patients with KMT2A/MLL-R$^+$ B-ALL vs. Normal Hematopoietic Cells.

Probeset	Fold Change (Ped ALL MLL-R$^+$ /Normal)	Linear Contrast (P-value)
FLT3_206674_at	22.38	$<1 \times 10^{-8}$
BLK_206255_at	4.38	$<1 \times 10^{-8}$
BLK_210934_at	1.79	$<1 \times 10^{-8}$
PTK2_1559529_at	1.59	6.2×10^{-6}
BTK_205504_at	1.49	9.9×10^{-5}
TEC_206301_at	1.45	2.7×10^{-4}
SYK_244023_at	1.21	6.8×10^{-2}
SRC_1558210_at	1.12	3.1×10^{-1}
ERBB3_1563252_at	1.10	3.5×10^{-1}
PTK2_207821_s_at	1.06	5.7×10^{-1}
ERBB3_202454_s_at	1.05	6.4×10^{-1}
JAK3_211109_at	1.03	8.0×10^{-1}
SRC_1565082_x_at	1.01	9.2×10^{-1}
JAK3_207187_at	1	9.6×10^{-1}
ERBB2_234354_x_at	1	9.8×10^{-1}
ERBB2_216836_s_at	0.99	9.3×10^{-1}
ERBB3_1563253_s_at	0.99	9.1×10^{-1}
ERBB1_224999_at	0.98	8.9×10^{-1}
PTK2_241453_at	0.98	8.2×10^{-1}
ERBB3_215638_at	0.97	7.4×10^{-1}
SRC_1565080_at	0.97	7.5×10^{-1}
ERBB1_201983_s_at	0.95	6.4×10^{-1}
JAK1_240613_at	0.95	5.9×10^{-1}
ERBB3_226213_at	0.94	5.5×10^{-1}
SRC_221284_s_at	0.94	5.5×10^{-1}
ERBB1_201984_s_at	0.93	5.3×10^{-1}
ERBB1_1565484_x_at	0.93	5.2×10^{-1}
JAK2_1562031_at	0.93	5.1×10^{-1}
ERBB1_211551_at	0.92	4.4×10^{-1}
MERTK_211912_at	0.92	4.4×10^{-1}
ERBB1_210984_x_at	0.88	2.3×10^{-1}
ERBB1_211607_x_at	0.87	2.0×10^{-1}
JAK3_211108_s_at	0.86	1.4×10^{-1}
ERBB2_210930_s_at	0.86	1.3×10^{-1}
ERBB1_211550_at	0.86	1.5×10^{-1}
SRC_213324_at	0.85	1.3×10^{-1}
ERBB1_1565483_at	0.84	1.1×10^{-1}
PTK2_208820_at	0.84	8.0×10^{-2}
SRC_221281_at	0.8	4.4×10^{-2}
MERTK_211913_s_at	0.8	4.1×10^{-2}
SRC_1558211_s_at	0.8	4.1×10^{-2}
JAK1_1552610_a_at	0.8	2.9×10^{-2}
SYK_209269_s_at	0.75	4.3×10^{-3}
JAK1_239695_at	0.73	2.6×10^{-3}
JAK2_205842_s_at	0.66	1.5×10^{-4}
LYN_210754_s_at	0.63	2.2×10^{-5}
JAK1_201648_at	0.61	1.9×10^{-6}
JAK2_205841_at	0.59	1.4×10^{-6}
TYK2_205546_s_at	0.53	$<1 \times 10^{-8}$
MERTK_206028_s_at	0.52	$<1 \times 10^{-8}$
SYK_207540_s_at	0.48	$<1 \times 10^{-8}$
FYN_216033_s_at	0.45	$<1 \times 10^{-8}$
FYN_212486_s_at	0.44	$<1 \times 10^{-8}$
LYN_202626_s_at	0.41	$<1 \times 10^{-8}$
SYK_226068_at	0.4	$<1 \times 10^{-8}$
LCK_204890_s_at	0.38	$<1 \times 10^{-8}$
FYN_210105_s_at	0.32	$<1 \times 10^{-8}$
JAK3_227677_at	0.26	$<1 \times 10^{-8}$
JAK1_1552611_a_at	0.21	$<1 \times 10^{-8}$
HCK_208018_s_at	0.20	$<1 \times 10^{-8}$
BMX_206464_at	0.20	$<1 \times 10^{-8}$
LCK_204891_s_at	0.20	$<1 \times 10^{-8}$
LYN_202625_at	0.16	$<1 \times 10^{-8}$
FGR_208438_s_at	0.02	$<1 \times 10^{-8}$

Table S3. Gene Expression Levels for Tyrosine Kinases in Leukemic Cells from Adult Patients with KMT2A/MLL-R^{+} B-ALL vs. Normal Hematopoietic Cells

Probeset	Fold Change (Adult ALL MLL-R^{+} /Normal)	Linear Contrast (P-value)
BLK_206255_at	21.79	<1 x 10^{-8}
FLT3_206674_at	21.00	<1 x 10^{-8}
BLK_210934_at	2.88	<1 x 10^{-8}
BTK_205504_at	2.43	<1 x 10^{-8}
TEC_206301_at	1.51	<1 x 10^{-8}
PTK2_208820_at	1.5	<1 x 10^{-8}
ERBB2_216836_s_at	1.38	7.3 x 10^{-6}
ERBB1_1565483_at	1.26	2.7 x 10^{-3}
PTK2_1559529_at	1.24	3.2 x 10^{-3}
ERBB1_1565484_x_at	1.22	1.2 x 10^{-2}
PTK2_241453_at	1.21	8.3 x 10^{-3}
PTK2_207821_s_at	1.19	1.6 x 10^{-2}
SRC_213324_at	1.17	4.3 x 10^{-2}
JAK1_240613_at	1.12	1.1 x 10^{-1}
TYK2_205546_s_at	1.08	2.6 x 10^{-1}
JAK1_239695_at	1.08	3.1 x 10^{-1}
ERBB1_211550_at	1.05	5.2 x 10^{-1}
JAK3_211109_at	1.04	5.7 x 10^{-1}
MERTK_211912_at	1.04	6.2 x 10^{-1}
ERBB2_234354_x_at	1.04	5.9 x 10^{-1}
JAK3_207187_at	1.04	6.1 x 10^{-1}
SRC_221281_at	1.03	7.0 x 10^{-1}
ERBB3_215638_at	1.02	7.4 x 10^{-1}
ERBB1_201983_s_at	1.01	8.8 x 10^{-1}
ERBB1_224999_at	1.01	8.9 x 10^{-1}
JAK2_1562031_at	1	9.7 x 10^{-1}
SRC_1558210_at	1	9.8 x 10^{-1}
SRC_1565080_at	0.99	9.2 x 10^{-1}
SRC_1565082_x_at	0.98	8.3 x 10^{-1}
ERBB1_201984_s_at	0.98	7.7 x 10^{-1}
ERBB1_211551_at	0.97	6.6 x 10^{-1}
ERBB3_1563253_s_at	0.96	6.2 x 10^{-1}
SRC_1558211_s_at	0.96	6.4 x 10^{-1}
ERBB1_210984_x_at	0.96	6.3 x 10^{-1}
SYK_244023_at	0.96	5.9 x 10^{-1}
ERBB3_202454_s_at	0.96	5.4 x 10^{-1}
ERBB3_226213_at	0.93	3.5 x 10^{-1}
JAK1_1552611_a_at	0.93	3.4 x 10^{-1}
ERBB3_1563252_at	0.93	3.0 x 10^{-1}
ERBB2_210930_s_at	0.93	3.0 x 10^{-1}
ERBB1_211607_x_at	0.93	3.3 x 10^{-1}
SRC_221284_s_at	0.93	3.3 x 10^{-1}
JAK3_227677_at	0.92	2.3 x 10^{-1}
SYK_207540_s_at	0.9	1.6 x 10^{-1}
JAK3_211108_s_at	0.9	1.6 x 10^{-1}
MERTK_211913_s_at	0.88	9.8 x 10^{-2}
SYK_226068_at	0.86	4.2 x 10^{-2}
JAK1_201648_at	0.8	2.0 x 10^{-3}
SYK_209269_s_at	0.66	<1 x 10^{-8}
JAK2_205841_at	0.64	<1 x 10^{-8}
LYN_202626_s_at	0.59	<1 x 10^{-8}
MERTK_206028_s_at	0.55	<1 x 10^{-8}
JAK1_1552610_a_at	0.55	<1 x 10^{-8}
LYN_202625_at	0.55	<1 x 10^{-8}
FYN_212486_s_at	0.54	<1 x 10^{-8}
JAK2_205842_s_at	0.53	<1 x 10^{-8}
FYN_210105_s_at	0.49	<1 x 10^{-8}
LCK_204890_s_at	0.47	<1 x 10^{-8}
HCK_208018_s_at	0.45	<1 x 10^{-8}
LYN_210754_s_at	0.45	<1 x 10^{-8}
FYN_216033_s_at	0.43	<1 x 10^{-8}
LCK_204891_s_at	0.43	<1 x 10^{-8}
BMX_206464_at	0.21	<1 x 10^{-8}
FGR_208438_s_at	0.07	<1 x 10^{-8}

Table S4. Gene Expression Levels for Tyrosine Kinases in Leukemic Cells from Infants with KMT2A/MLL-R$^+$ B-ALL vs Infants with KMT2A/MLL-R- (MLL germline/WT) ALL.

Probeset	Fold Change (Infant ALL MLL-R$^+$ / MLL-R$^-$)	Linear Contrast (P-value)
FLT3_206674_at	11.23	$<1 \times 10^{-8}$
BLK_206255_at	3.98	$<1 \times 10^{-8}$
HCK_208018_s_at	2.46	$<1 \times 10^{-8}$
BTK_205504_at	1.99	3.0×10^{-7}
LYN_202626_s_at	1.92	6.0×10^{-7}
JAK3_227677_at	1.88	2.6×10^{-6}
LYN_210754_s_at	1.88	1.3×10^{-6}
BLK_210934_at	1.88	2.8×10^{-6}
LYN_202625_at	1.85	2.5×10^{-6}
PTK2_208820_at	1.67	1.4×10^{-4}
JAK2_205842_s_at	1.63	1.7×10^{-4}
TYK2_205546_s_at	1.59	5.8×10^{-4}
JAK2_205841_at	1.48	2.5×10^{-3}
JAK2_1562031_at	1.47	3.3×10^{-3}
SYK_226068_at	1.46	4.5×10^{-3}
SYK_207540_s_at	1.45	5.3×10^{-3}
PTK2_207821_s_at	1.43	8.0×10^{-3}
TEC_206301_at	1.33	3.6×10^{-2}
FGR_208438_s_at	1.31	3.6×10^{-2}
SYK_209269_s_at	1.23	1.2×10^{-1}
SRC_213324_at	1.16	2.6×10^{-1}
ERBB2_216836_s_at	1.14	3.4×10^{-1}
JAK1_1552610_a_at	1.11	4.2×10^{-1}
PTK2_241453_at	1.09	5.0×10^{-1}
SYK_244023_at	1.09	5.2×10^{-1}
ERBB1_1565484_x_at	1.07	6.2×10^{-1}
JAK1_1552611_a_at	1.05	7.0×10^{-1}
PTK2_1559529_at	1.05	7.0×10^{-1}
ERBB3_1563253_s_at	1.05	7.4×10^{-1}
ERBB1_1565483_at	1.03	8.0×10^{-1}
ERBB1_210984_x_at	1.03	8.2×10^{-1}
JAK3_211108_s_at	1.03	8.3×10^{-1}
FYN_216033_s_at	1.02	8.7×10^{-1}
ERBB1_211550_at	1.02	8.8×10^{-1}
SRC_1558211_s_at	1.02	9.1×10^{-1}
FYN_212486_s_at	1.01	9.3×10^{-1}
JAK1_239695_at	1	9.9×10^{-1}
SRC_1565082_x_at	1	9.9×10^{-1}
ERBB2_234354_x_at	1	9.8×10^{-1}
ERBB1_201983_s_at	1	9.7×10^{-1}
JAK3_207187_at	1	9.7×10^{-1}
JAK1_201648_at	0.99	9.6×10^{-1}
ERBB3_226213_at	0.99	9.5×10^{-1}
SRC_1565080_at	0.99	9.4×10^{-1}
FYN_210105_s_at	0.99	9.4×10^{-1}
ERBB2_210930_s_at	0.99	9.3×10^{-1}
ERBB3_215638_at	0.99	9.2×10^{-1}
MERTK_211913_s_at	0.98	9.1×10^{-1}
ERBB3_202454_s_at	0.98	9.0×10^{-1}
ERBB1_201984_s_at	0.98	8.9×10^{-1}
SRC_221281_at	0.97	8.2×10^{-1}
SRC_1558210_at	0.96	7.8×10^{-1}
SRC_221284_s_at	0.96	7.7×10^{-1}
ERBB1_224999_at	0.96	7.6×10^{-1}
BMX_206464_at	0.96	7.5×10^{-1}
MERTK_211912_at	0.96	7.3×10^{-1}
ERBB3_1563252_at	0.95	7.3×10^{-1}
JAK3_211109_at	0.95	7.2×10^{-1}
ERBB1_211551_at	0.95	7.1×10^{-1}
JAK1_240613_at	0.94	6.6×10^{-1}
ERBB1_211607_x_at	0.93	6.0×10^{-1}
MERTK_206028_s_at	0.88	3.2×10^{-1}
LCK_204890_s_at	0.85	2.2×10^{-1}
LCK_204891_s_at	0.85	2.0×10^{-1}

Table S5. Gene Expression Levels for Tyrosine Kinases in Leukemic Cells from Pediatric Patients with KMT2A/MLL-R$^+$ B-ALL vs Other Types of ALL without KMT2A/MLL Rerrangements.

Probeset	Fold Change (Ped ALL MLL-R$^+$ / Other)	Linear Contrast (P-value)
FLT3_206674_at	8.65	$<1 \times 10^{-8}$
BLK_206255_at	3.11	$<1 \times 10^{-8}$
HCK_208018_s_at	2.64	$<1 \times 10^{-8}$
BTK_205504_at	2.03	$<1 \times 10^{-8}$
LYN_202626_s_at	2	$<1 \times 10^{-8}$
SYK_207540_s_at	1.91	1.0×10^{-7}
LYN_210754_s_at	1.81	1.0×10^{-6}
LYN_202625_at	1.77	2.5×10^{-6}
SYK_226068_at	1.75	5.2×10^{-6}
JAK2_205842_s_at	1.60	1.1×10^{-4}
BLK_210934_at	1.46	2.2×10^{-3}
SYK_244023_at	1.35	1.3×10^{-2}
JAK3_227677_at	1.35	1.4×10^{-2}
TYK2_205546_s_at	1.35	1.4×10^{-2}
SYK_209269_s_at	1.27	5.5×10^{-2}
JAK2_205841_at	1.26	5.3×10^{-2}
TEC_206301_at	1.25	7.2×10^{-2}
JAK2_1562031_at	1.24	7.1×10^{-2}
JAK1_201648_at	1.11	3.9×10^{-1}
FGR_208438_s_at	1.1	4.1×10^{-1}
SRC_221284_s_at	1	9.8×10^{-1}
MERTK_211912_at	0.99	9.6×10^{-1}
JAK3_211108_s_at	0.99	9.3×10^{-1}
BMX_206464_at	0.98	8.8×10^{-1}
JAK3_211109_at	0.98	8.5×10^{-1}
JAK1_1552610_a_at	0.97	8.2×10^{-1}
ERBB3_1563252_at	0.97	8.0×10^{-1}
ERBB1_210984_x_at	0.97	7.9×10^{-1}
SRC_1558210_at	0.97	7.8×10^{-1}
ERBB1_201983_s_at	0.96	7.4×10^{-1}
JAK3_207187_at	0.96	7.2×10^{-1}
ERBB3_215638_at	0.96	7.1×10^{-1}
SRC_1565080_at	0.95	7.0×10^{-1}
SRC_221281_at	0.95	6.8×10^{-1}
ERBB1_211551_at	0.95	6.5×10^{-1}
MERTK_211913_s_at	0.95	6.4×10^{-1}
SRC_1565082_x_at	0.94	6.3×10^{-1}
MERTK_206028_s_at	0.94	6.1×10^{-1}
ERBB1_211607_x_at	0.94	6.0×10^{-1}
ERBB2_234354_x_at	0.93	5.7×10^{-1}
ERBB2_210930_s_at	0.93	5.5×10^{-1}
ERBB1_224999_at	0.92	5.1×10^{-1}
ERBB3_202454_s_at	0.92	4.9×10^{-1}
FYN_212486_s_at	0.92	4.8×10^{-1}
ERBB3_1563253_s_at	0.92	4.7×10^{-1}
SRC_1558211_s_at	0.9	4.1×10^{-1}
ERBB1_211550_at	0.89	3.6×10^{-1}
ERBB1_201984_s_at	0.89	3.3×10^{-1}
PTK2_241453_at	0.88	2.9×10^{-1}
ERBB2_216836_s_at	0.87	2.5×10^{-1}
ERBB3_226213_at	0.84	1.6×10^{-1}
ERBB1_1565484_x_at	0.84	1.4×10^{-1}
JAK1_1552611_a_at	0.83	1.4×10^{-1}
LCK_204890_s_at	0.82	9.4×10^{-2}
SRC_213324_at	0.8	7.1×10^{-2}
ERBB1_1565483_at	0.8	6.7×10^{-2}
JAK1_240613_at	0.8	7.1×10^{-2}
FYN_210105_s_at	0.79	5.4×10^{-2}
FYN_216033_s_at	0.78	3.6×10^{-2}
PTK2_1559529_at	0.77	3.6×10^{-2}
LCK_204891_s_at	0.71	4.5×10^{-3}
PTK2_207821_s_at	0.7	3.7×10^{-3}
JAK1_239695_at	0.67	9.4×10^{-4}
PTK2_208820_at	0.62	8.4×10^{-5}

Table S6. Gene Expression Levels for Tyrosine Kinases in Leukemic Cells from Adult Patients with KMT2A/MLL-R+ B-ALL vs Other Types of ALL without KMT2A/MLL Rerrangements

Probeset	Fold Change (Adult ALL MLL-R+ / Other)	Linear Contrast (P-value)
FLT3_206674_at	5.69	$<1 \times 10^{-8}$
BLK_206255_at	3.95	$<1 \times 10^{-8}$
HCK_208018_s_at	2.21	$<1 \times 10^{-8}$
BLK_210934_at	1.86	$<1 \times 10^{-8}$
LYN_202625_at	1.71	$<1 \times 10^{-8}$
LYN_202626_s_at	1.64	$<1 \times 10^{-8}$
LYN_210754_s_at	1.44	1.1×10^{-5}
BTK_205504_at	1.43	1.1×10^{-5}
TEC_206301_at	1.24	8.7×10^{-3}
JAK3_227677_at	1.23	9.5×10^{-3}
SYK_207540_s_at	1.16	6.0×10^{-2}
JAK2_205841_at	1.16	7.4×10^{-2}
SYK_226068_at	1.16	7.1×10^{-2}
FGR_208438_s_at	1.15	9.4×10^{-2}
ERBB1_1565483_at	1.1	2.5×10^{-1}
JAK2_205842_s_at	1.09	3.0×10^{-1}
ERBB1_210984_x_at	1.06	4.9×10^{-1}
SRC_221284_s_at	1.06	5.1×10^{-1}
ERBB1_1565484_x_at	1.05	5.5×10^{-1}
JAK2_1562031_at	1.05	5.9×10^{-1}
JAK3_211109_at	1.05	5.8×10^{-1}
ERBB3_1563252_at	1.04	6.2×10^{-1}
ERBB1_224999_at	1.04	6.6×10^{-1}
ERBB1_201983_s_at	1.03	6.9×10^{-1}
ERBB1_211551_at	1.03	7.0×10^{-1}
ERBB3_215638_at	1.03	7.3×10^{-1}
ERBB2_234354_x_at	1.02	7.7×10^{-1}
ERBB1_211550_at	1.02	7.9×10^{-1}
SRC_1565080_at	1.02	7.9×10^{-1}
TYK2_205546_s_at	1.02	8.3×10^{-1}
SRC_1558210_at	1.01	9.1×10^{-1}
ERBB2_216836_s_at	1.01	9.3×10^{-1}
ERBB1_211607_x_at	1.01	9.4×10^{-1}
SRC_1565082_x_at	1	9.7×10^{-1}
MERTK_211912_at	1	9.7×10^{-1}
ERBB2_210930_s_at	1	9.7×10^{-1}
ERBB3_1563253_s_at	1	9.6×10^{-1}
JAK3_211108_s_at	1	9.6×10^{-1}
SRC_221281_at	1	9.5×10^{-1}
ERBB1_201984_s_at	0.99	9.5×10^{-1}
SRC_1558211_s_at	0.99	9.2×10^{-1}
JAK3_207187_at	0.99	8.6×10^{-1}
BMX_206464_at	0.98	7.7×10^{-1}
SYK_209269_s_at	0.97	7.1×10^{-1}
MERTK_211913_s_at	0.97	7.0×10^{-1}
SRC_213324_at	0.96	6.2×10^{-1}
ERBB3_202454_s_at	0.96	5.8×10^{-1}
ERBB3_226213_at	0.91	2.5×10^{-1}
PTK2_241453_at	0.88	1.2×10^{-1}
MERTK_206028_s_at	0.84	4.2×10^{-2}
SYK_244023_at	0.81	7.9×10^{-3}
PTK2_207821_s_at	0.76	8.1×10^{-4}
JAK1_201648_at	0.75	3.4×10^{-4}
JAK1_1552611_a_at	0.74	2.1×10^{-4}
PTK2_1559529_at	0.73	1.0×10^{-4}
FYN_216033_s_at	0.69	1.0×10^{-5}
JAK1_240613_at	0.69	3.0×10^{-6}
JAK1_1552610_a_at	0.64	$<1 \times 10^{-8}$
FYN_212486_s_at	0.64	1.0×10^{-7}
FYN_210105_s_at	0.62	$<1 \times 10^{-8}$
JAK1_239695_at	0.62	$<1 \times 10^{-8}$
PTK2_208820_at	0.57	$<1 \times 10^{-8}$
LCK_204890_s_at	0.55	$<1 \times 10^{-8}$
LCK_204891_s_at	0.38	$<1 \times 10^{-8}$

Table S7. Gene Expression Levels for Tyrosine Kinases in Leukemic Cells from Pediatric Patients with KMT2A/MLL-R$^+$ AML vs. Normal Hematopoietic Cells

Probeset	Fold Change (Ped AML MLL-R$^+$/Normal)	Linear Contrast (P-value)
FLT3_206674_at	7.54	$<1 \times 10^{-8}$
TEC_206301_at	2.20	$<1 \times 10^{-8}$
SRC_213324_at	1.64	5.6×10^{-9}
JAK2_1562031_at	1.59	4.8×10^{-8}
LYN_210754_s_at	1.58	6.1×10^{-8}
BTK_205504_at	1.47	5.4×10^{-6}
JAK2_205841_at	1.41	5.9×10^{-5}
ERBB3_1563253_s_at	1.39	9.7×10^{-5}
PTK2_1559529_at	1.34	6.2×10^{-4}
SRC_1565082_x_at	1.30	1.7×10^{-3}
SYK_244023_at	1.30	2.1×10^{-3}
SYK_209269_s_at	1.29	2.5×10^{-3}
ERBB1_1565484_x_at	1.29	3.0×10^{-3}
JAK2_205842_s_at	1.24	1.1×10^{-2}
JAK3_207187_at	1.23	1.4×10^{-2}
PTK2_241453_at	1.21	2.6×10^{-2}
MERTK_206028_s_at	1.21	2.8×10^{-2}
ERBB1_201984_s_at	1.20	3.4×10^{-2}
ERBB3_226213_at	1.19	4.4×10^{-2}
ERBB2_216836_s_at	1.17	6.2×10^{-2}
LYN_202626_s_at	1.17	6.5×10^{-2}
SRC_1565080_at	1.16	9.0×10^{-2}
MERTK_211913_s_at	1.15	1.0×10^{-1}
PTK2_207821_s_at	1.13	1.5×10^{-1}
ERBB1_210984_x_at	1.13	1.6×10^{-1}
ERBB1_1565483_at	1.12	1.9×10^{-1}
SRC_1558211_s_at	1.12	2.0×10^{-1}
ERBB1_211550_at	1.08	3.8×10^{-1}
JAK1_239695_at	1.07	4.0×10^{-1}
ERBB1_201983_s_at	1.07	4.1×10^{-1}
ERBB3_202454_s_at	1.07	4.1×10^{-1}
ERBB1_224999_at	1.06	4.9×10^{-1}
ERBB1_211607_x_at	1.05	5.9×10^{-1}
SRC_1558210_at	1.03	7.3×10^{-1}
ERBB3_1563252_at	1.03	7.4×10^{-1}
ERBB1_211551_at	1.02	7.8×10^{-1}
LYN_202625_at	1.01	8.9×10^{-1}
ERBB2_234354_x_at	1.01	9.4×10^{-1}
JAK1_240613_at	1	9.6×10^{-1}
BLK_210934_at	0.96	6.0×10^{-1}
MERTK_211912_at	0.95	5.4×10^{-1}
ERBB3_215638_at	0.92	3.2×10^{-1}
JAK3_211109_at	0.9	1.9×10^{-1}
TYK2_205546_s_at	0.89	1.6×10^{-1}
JAK3_211108_s_at	0.85	5.7×10^{-2}
ERBB2_210930_s_at	0.82	2.0×10^{-2}
SYK_226068_at	0.82	1.7×10^{-2}
PTK2_208820_at	0.81	1.3×10^{-2}
SRC_221284_s_at	0.8	1.0×10^{-2}
SRC_221281_at	0.75	6.1×10^{-4}
JAK1_201648_at	0.72	1.5×10^{-4}
HCK_208018_s_at	0.71	4.8×10^{-5}
SYK_207540_s_at	0.68	7.5×10^{-6}
JAK1_1552611_a_at	0.66	8.4×10^{-7}
JAK1_1552610_a_at	0.65	5.9×10^{-7}
BLK_206255_at	0.58	$<1 \times 10^{-8}$
FYN_212486_s_at	0.58	$<1 \times 10^{-8}$
LCK_204890_s_at	0.56	$<1 \times 10^{-8}$
JAK3_227677_at	0.54	$<1 \times 10^{-8}$
FYN_216033_s_at	0.53	$<1 \times 10^{-8}$
FYN_210105_s_at	0.47	$<1 \times 10^{-8}$
FGR_208438_s_at	0.33	$<1 \times 10^{-8}$
BMX_206464_at	0.32	$<1 \times 10^{-8}$
LCK_204891_s_at	0.32	$<1 \times 10^{-8}$

Table S8. Gene Expression Levels for Tyrosine Kinases in Leukemic Cells from Adult Patients with KMT2A/MLL-R^{+} AML vs. Normal Hematopoietic Cells

Probeset	Fold Change (Adult AML MLL-R^{+} /Normal)	Linear Contrast (P-value)
FLT3_206674_at	8.28	$<1 \times 10^{-8}$
TEC_206301_at	1.73	$<1 \times 10^{-8}$
TYK2_205546_s_at	1.39	8.5×10^{-5}
BTK_205504_at	1.33	6.8×10^{-4}
MERTK_206028_s_at	1.26	2.1×10^{-2}
MERTK_211913_s_at	1.25	2.6×10^{-2}
LYN_202626_s_at	1.21	5.3×10^{-2}
JAK2_1562031_at	1.19	7.7×10^{-2}
ERBB1_1565483_at	1.18	9.3×10^{-2}
LYN_202625_at	1.17	1.2×10^{-1}
ERBB1_1565484_x_at	1.15	1.7×10^{-1}
SRC_213324_at	1.14	1.8×10^{-1}
LYN_210754_s_at	1.12	2.5×10^{-1}
ERBB3_226213_at	1.11	2.2×10^{-1}
MERTK_211912_at	1.08	4.4×10^{-1}
ERBB1_211550_at	1.08	4.5×10^{-1}
ERBB3_202454_s_at	1.07	4.0×10^{-1}
JAK3_211109_at	1.07	4.5×10^{-1}
SRC_221281_at	1.06	5.6×10^{-1}
JAK3_207187_at	1.06	5.2×10^{-1}
ERBB1_201983_s_at	1.05	6.4×10^{-1}
SRC_1558210_at	1.05	6.5×10^{-1}
SRC_1565080_at	1.04	7.2×10^{-1}
ERBB2_234354_x_at	1.02	8.0×10^{-1}
SRC_1565082_x_at	1.01	9.6×10^{-1}
ERBB3_215638_at	1	9.8×10^{-1}
ERBB1_201984_s_at	1	9.9×10^{-1}
ERBB1_224999_at	1	9.9×10^{-1}
JAK3_211108_s_at	1	9.6×10^{-1}
ERBB1_211551_at	0.99	9.3×10^{-1}
ERBB1_210984_x_at	0.98	8.2×10^{-1}
ERBB1_211607_x_at	0.98	8.1×10^{-1}
PTK2_241453_at	0.98	7.7×10^{-1}
ERBB2_210930_s_at	0.97	7.4×10^{-1}
ERBB3_1563252_at	0.97	7.3×10^{-1}
PTK2_1559529_at	0.97	7.2×10^{-1}
SRC_1558211_s_at	0.96	6.7×10^{-1}
SYK_207540_s_at	0.95	5.8×10^{-1}
BLK_210934_at	0.94	4.3×10^{-1}
ERBB2_216836_s_at	0.91	2.6×10^{-1}
HCK_208018_s_at	0.91	3.3×10^{-1}
JAK1_240613_at	0.9	2.3×10^{-1}
SYK_226068_at	0.9	2.1×10^{-1}
SRC_221284_s_at	0.9	2.8×10^{-1}
JAK2_205842_s_at	0.9	2.7×10^{-1}
ERBB3_1563253_s_at	0.88	1.2×10^{-1}
JAK2_205841_at	0.82	5.0×10^{-2}
PTK2_207821_s_at	0.81	1.0×10^{-2}
SYK_209269_s_at	0.77	2.1×10^{-3}
JAK1_1552611_a_at	0.71	6.5×10^{-5}
JAK3_227677_at	0.7	2.4×10^{-5}
JAK1_239695_at	0.66	8.0×10^{-7}
JAK1_201648_at	0.65	3.0×10^{-7}
BLK_206255_at	0.64	1.0×10^{-7}
SYK_244023_at	0.61	$<1 \times 10^{-8}$
FGR_208438_s_at	0.58	$<1 \times 10^{-8}$
FYN_210105_s_at	0.54	$<1 \times 10^{-8}$
FYN_216033_s_at	0.54	$<1 \times 10^{-8}$
JAK1_1552610_a_at	0.53	$<1 \times 10^{-8}$
FYN_212486_s_at	0.44	$<1 \times 10^{-8}$
PTK2_208820_at	0.42	$<1 \times 10^{-8}$
LCK_204890_s_at	0.38	$<1 \times 10^{-8}$
BMX_206464_at	0.26	$<1 \times 10^{-8}$
LCK_204891_s_at	0.25	$<1 \times 10^{-8}$

Table S9. Gene Expression Levels for Tyrosine Kinases in Leukemic Cells from Pediatric Patients with KMT2A/MLL-R^{+} AML vs. Other subsets of AML without KMT2A/MLL Rearrangements

Probeset	Fold Change (Ped AML MLL-R^{+} / Other)	Linear Contrast (P-value)
FGR_208438_s_at	4.31	$<1 \times 10^{-8}$
SYK_207540_s_at	4.01	$<1 \times 10^{-8}$
HCK_208018_s_at	3.97	$<1 \times 10^{-8}$
SYK_226068_at	3.26	$<1 \times 10^{-8}$
JAK1_1552611_a_at	2.69	$<1 \times 10^{-8}$
SYK_209269_s_at	2.31	$<1 \times 10^{-8}$
JAK2_205841_at	2.26	$<1 \times 10^{-8}$
JAK2_205842_s_at	2.16	$<1 \times 10^{-8}$
LYN_202625_at	1.74	$<1 \times 10^{-8}$
LYN_202626_s_at	1.69	$<1 \times 10^{-8}$
LYN_210754_s_at	1.67	$<1 \times 10^{-8}$
JAK1_239695_at	1.66	$<1 \times 10^{-8}$
FLT3_206674_at	1.65	$<1 \times 10^{-8}$
PTK2_208820_at	1.62	$<1 \times 10^{-8}$
FYN_212486_s_at	1.61	$<1 \times 10^{-8}$
LCK_204891_s_at	1.54	$<1 \times 10^{-8}$
JAK2_1562031_at	1.52	$<1 \times 10^{-8}$
TYK2_205546_s_at	1.51	$<1 \times 10^{-8}$
BTK_205504_at	1.42	1.4×10^{-7}
SYK_244023_at	1.34	1.2×10^{-5}
JAK1_201648_at	1.34	1.4×10^{-5}
BLK_206255_at	1.3	9.5×10^{-5}
FYN_210105_s_at	1.28	1.9×10^{-4}
LCK_204890_s_at	1.23	2.0×10^{-3}
MERTK_206028_s_at	1.16	2.8×10^{-2}
JAK1_1552610_a_at	1.13	5.9×10^{-2}
JAK1_240613_at	1.13	7.3×10^{-2}
PTK2_1559529_at	1.12	9.3×10^{-2}
BMX_206464_at	1.1	1.5×10^{-1}
MERTK_211913_s_at	1.09	1.9×10^{-1}
SRC_221284_s_at	1.06	3.9×10^{-1}
PTK2_241453_at	1.05	4.4×10^{-1}
SRC_213324_at	1.04	5.8×10^{-1}
JAK3_227677_at	1.01	8.3×10^{-1}
ERBB2_234354_x_at	1.01	8.4×10^{-1}
TEC_206301_at	1.01	9.1×10^{-1}
PTK2_207821_s_at	1	1.0×10^{0}
SRC_1558211_s_at	1	9.8×10^{-1}
FYN_216033_s_at	1	9.8×10^{-1}
JAK3_211109_at	0.98	7.7×10^{-1}
ERBB2_210930_s_at	0.98	7.3×10^{-1}
ERBB3_1563253_s_at	0.97	6.2×10^{-1}
SRC_1558210_at	0.96	5.7×10^{-1}
JAK3_211108_s_at	0.95	4.2×10^{-1}
BLK_210934_at	0.95	4.2×10^{-1}
ERBB2_216836_s_at	0.93	3.1×10^{-1}
ERBB3_1563252_at	0.93	2.7×10^{-1}
JAK3_207187_at	0.92	2.1×10^{-1}
ERBB1_224999_at	0.9	1.3×10^{-1}
SRC_221281_at	0.89	8.6×10^{-2}
ERBB1_211551_at	0.89	8.0×10^{-2}
ERBB3_202454_s_at	0.87	3.7×10^{-2}
ERBB3_215638_at	0.85	1.8×10^{-2}
ERBB1_201984_s_at	0.85	1.7×10^{-2}
ERBB1_211550_at	0.85	1.6×10^{-2}
ERBB3_226213_at	0.85	1.5×10^{-2}
SRC_1565082_x_at	0.83	6.9×10^{-3}
ERBB1_201983_s_at	0.8	7.9×10^{-4}
ERBB1_210984_x_at	0.79	4.4×10^{-4}
ERBB1_211607_x_at	0.76	4.4×10^{-5}
SRC_1565080_at	0.76	3.7×10^{-5}
MERTK_211912_at	0.72	1.1×10^{-6}
ERBB1_1565484_x_at	0.55	$<1 \times 10^{-8}$
ERBB1_1565483_at	0.51	$<1 \times 10^{-8}$

Table S10. Gene Expression Levels for Tyrosine Kinases in Leukemic Cells from Adult Patients with KMT2A/MLL-R$^+$ AML vs. Other subsets of AML without KMT2A/MLL Rearrangements.

Probeset	Fold Change (Adult AML MLL-R$^+$ / Other)	Linear Contrast (P-value)
FGR_208438_s_at	2.46	$<1 \times 10^{-8}$
HCK_208018_s_at	2.10	$<1 \times 10^{-8}$
TYK2_205546_s_at	1.29	1.2×10^{-3}
SYK_226068_at	1.20	2.0×10^{-2}
SYK_207540_s_at	1.19	2.7×10^{-2}
BLK_206255_at	1.18	4.2×10^{-2}
LYN_202625_at	1.12	2.2×10^{-1}
FLT3_206674_at	1.12	2.3×10^{-1}
LYN_202626_s_at	1.12	2.4×10^{-1}
JAK2_1562031_at	1.11	2.7×10^{-1}
ERBB3_226213_at	1.09	2.9×10^{-1}
LYN_210754_s_at	1.09	3.7×10^{-1}
ERBB1_1565483_at	1.07	4.7×10^{-1}
ERBB3_202454_s_at	1.06	4.9×10^{-1}
SYK_209269_s_at	1.06	4.9×10^{-1}
JAK2_205842_s_at	1.06	5.7×10^{-1}
JAK2_205841_at	1.05	5.8×10^{-1}
ERBB1_211550_at	1.04	6.6×10^{-1}
MERTK_211912_at	1.04	6.7×10^{-1}
BTK_205504_at	1.04	6.5×10^{-1}
SRC_221281_at	1.03	7.6×10^{-1}
SRC_213324_at	1.03	7.9×10^{-1}
ERBB1_201983_s_at	1.02	8.0×10^{-1}
SRC_1558210_at	1.02	8.1×10^{-1}
SRC_1565080_at	1.02	8.6×10^{-1}
ERBB1_1565484_x_at	1.02	8.6×10^{-1}
ERBB2_210930_s_at	1.01	8.5×10^{-1}
JAK3_211109_at	1.01	8.6×10^{-1}
LCK_204890_s_at	1.01	9.1×10^{-1}
ERBB2_234354_x_at	1.01	9.1×10^{-1}
ERBB1_210984_x_at	1.01	9.3×10^{-1}
ERBB1_211607_x_at	1.01	9.6×10^{-1}
ERBB3_215638_at	1	9.5×10^{-1}
PTK2_241453_at	1	1.0×10^{0}
ERBB1_201984_s_at	0.99	9.5×10^{-1}
SRC_1565082_x_at	0.99	9.4×10^{-1}
ERBB1_211551_at	0.99	9.0×10^{-1}
TEC_206301_at	0.99	8.7×10^{-1}
ERBB1_224999_at	0.99	8.9×10^{-1}
ERBB3_1563252_at	0.98	8.4×10^{-1}
JAK3_207187_at	0.98	7.6×10^{-1}
SRC_221284_s_at	0.98	8.0×10^{-1}
SRC_1558211_s_at	0.97	7.5×10^{-1}
JAK3_211108_s_at	0.97	7.0×10^{-1}
BLK_210934_at	0.97	6.8×10^{-1}
MERTK_211913_s_at	0.96	6.8×10^{-1}
PTK2_1559529_at	0.95	5.3×10^{-1}
BMX_206464_at	0.95	4.9×10^{-1}
ERBB2_216836_s_at	0.94	4.1×10^{-1}
ERBB3_1563253_s_at	0.92	2.8×10^{-1}
JAK1_240613_at	0.89	1.6×10^{-1}
PTK2_207821_s_at	0.88	1.1×10^{-1}
LCK_204891_s_at	0.88	1.8×10^{-1}
SYK_244023_at	0.88	1.1×10^{-1}
FYN_216033_s_at	0.86	1.1×10^{-1}
FYN_210105_s_at	0.82	3.2×10^{-2}
JAK1_1552611_a_at	0.76	6.9×10^{-4}
JAK1_201648_at	0.75	2.6×10^{-4}
FYN_212486_s_at	0.75	2.0×10^{-3}
MERTK_206028_s_at	0.74	1.3×10^{-3}
PTK2_208820_at	0.73	6.5×10^{-5}
JAK1_1552610_a_at	0.71	1.4×10^{-5}
JAK1_239695_at	0.71	1.1×10^{-5}
JAK3_227677_at	0.55	$<1 \times 10^{-8}$

Table S11. Gene Expression Levels for Tyrosine Kinases in Leukemic Cells from Pediatric AML Patients with FLT3-ITD⁺ mutation vs Normal Hematopoietic Cells.

Probeset	Fold Change (Ped AML FLT3-ITD^{+} / Normal)	Linear Contrast (P-value)
FLT3_206674_at	9.01	$<1 \times 10^{-8}$
TEC_206301_at	2.58	$<1 \times 10^{-8}$
ERBB1_1565484_x_at	2.24	$<1 \times 10^{-8}$
ERBB1_1565483_at	1.97	$<1 \times 10^{-8}$
SRC_213324_at	1.58	1.0×10^{-7}
SRC_1565082_x_at	1.57	3.0×10^{-7}
SRC_1565080_at	1.49	4.7×10^{-6}
ERBB3_1563253_s_at	1.45	3.0×10^{-7}
ERBB3_226213_at	1.42	1.3×10^{-6}
ERBB1_210984_x_at	1.41	9.2×10^{-5}
ERBB1_211607_x_at	1.38	2.1×10^{-4}
ERBB1_201984_s_at	1.37	2.6×10^{-4}
ERBB1_201983_s_at	1.32	1.4×10^{-3}
JAK3_207187_at	1.32	1.5×10^{-4}
MERTK_211912_at	1.3	2.5×10^{-3}
BTK_205504_at	1.29	6.0×10^{-4}
ERBB3_202454_s_at	1.25	2.4×10^{-3}
ERBB1_211550_at	1.25	1.2×10^{-2}
ERBB2_216836_s_at	1.21	7.8×10^{-3}
ERBB1_224999_at	1.19	4.4×10^{-2}
SRC_1558211_s_at	1.14	1.4×10^{-1}
ERBB1_211551_at	1.13	1.6×10^{-1}
ERBB3_1563252_at	1.1	1.8×10^{-1}
SRC_1558210_at	1.09	3.5×10^{-1}
JAK2_1562031_at	1.08	3.5×10^{-1}
PTK2_1559529_at	1.07	3.4×10^{-1}
PTK2_241453_at	1.07	3.5×10^{-1}
MERTK_211913_s_at	1.07	4.4×10^{-1}
ERBB3_215638_at	1.07	3.8×10^{-1}
MERTK_206028_s_at	1.06	5.3×10^{-1}
ERBB2_234354_x_at	1	1.0×10^{0}
BLK_210934_at	1	9.8×10^{-1}
SYK_244023_at	0.95	4.9×10^{-1}
PTK2_207821_s_at	0.93	3.5×10^{-1}
JAK3_211109_at	0.89	1.3×10^{-1}
JAK3_211108_s_at	0.89	9.4×10^{-2}
JAK1_240613_at	0.85	2.7×10^{-2}
ERBB2_210930_s_at	0.84	1.7×10^{-2}
SRC_221281_at	0.84	4.0×10^{-2}
SRC_221284_s_at	0.76	2.1×10^{-3}
LYN_210754_s_at	0.75	1.1×10^{-3}
JAK2_205841_at	0.65	8.0×10^{-7}
TYK2_205546_s_at	0.64	$<1 \times 10^{-8}$
JAK2_205842_s_at	0.62	1.0×10^{-7}
SYK_209269_s_at	0.6	$<1 \times 10^{-8}$
JAK1_239695_at	0.59	$<1 \times 10^{-8}$
LYN_202626_s_at	0.56	$<1 \times 10^{-8}$
LYN_202625_at	0.51	$<1 \times 10^{-8}$
FYN_216033_s_at	0.51	$<1 \times 10^{-8}$
JAK1_1552610_a_at	0.5	$<1 \times 10^{-8}$
JAK1_201648_at	0.48	$<1 \times 10^{-8}$
JAK3_227677_at	0.47	$<1 \times 10^{-8}$
LCK_204890_s_at	0.41	$<1 \times 10^{-8}$
BLK_206255_at	0.38	$<1 \times 10^{-8}$
FYN_212486_s_at	0.37	$<1 \times 10^{-8}$
FYN_210105_s_at	0.36	$<1 \times 10^{-8}$
PTK2_208820_at	0.34	$<1 \times 10^{-8}$
BMX_206464_at	0.31	$<1 \times 10^{-8}$
SYK_226068_at	0.3	$<1 \times 10^{-8}$
HCK_208018_s_at	0.27	$<1 \times 10^{-8}$
JAK1_1552611_a_at	0.24	$<1 \times 10^{-8}$
SYK_207540_s_at	0.18	$<1 \times 10^{-8}$
LCK_204891_s_at	0.15	$<1 \times 10^{-8}$
FGR_208438_s_at	0.08	$<1 \times 10^{-8}$

Table S12. Gene Expression Levels for Tyrosine Kinases in Leukemic Cells from Pediatric AML Patients with FLT3-ITD$^+$ mutation vs FLT3-ITD$^-$.

Probeset	Fold Change (Ped AML FLT3-ITD$^+$ / FLT3-ITD$^-$)	Linear Contrast (P-value)
FLT3_206674_at	1.96	$<1 \times 10^{-8}$
BTK_205504_at	1.25	9.8×10^{-4}
TEC_206301_at	1.23	2.5×10^{-3}
HCK_208018_s_at	1.22	1.4×10^{-2}
BMX_206464_at	1.11	1.3×10^{-1}
SYK_226068_at	1.07	3.1×10^{-1}
FYN_212486_s_at	1.07	4.2×10^{-1}
FYN_210105_s_at	1.07	4.4×10^{-1}
JAK2_205842_s_at	1.05	5.2×10^{-1}
TYK2_205546_s_at	1.04	5.4×10^{-1}
MERTK_206028_s_at	1.04	6.3×10^{-1}
ERBB3_226213_at	1.03	6.2×10^{-1}
MERTK_211913_s_at	1.03	7.4×10^{-1}
ERBB3_202454_s_at	1.02	7.4×10^{-1}
SRC_1558210_at	1.02	8.1×10^{-1}
SRC_1558211_s_at	1.02	8.2×10^{-1}
ERBB1_224999_at	1.02	8.3×10^{-1}
JAK1_1552611_a_at	1.01	8.3×10^{-1}
ERBB1_211607_x_at	1.01	9.0×10^{-1}
JAK2_1562031_at	1.01	9.2×10^{-1}
MERTK_211912_at	1.01	9.5×10^{-1}
ERBB3_1563253_s_at	1	9.7×10^{-1}
SRC_213324_at	1	9.8×10^{-1}
SRC_221281_at	1	9.9×10^{-1}
ERBB2_210930_s_at	1	9.9×10^{-1}
SRC_1565082_x_at	1	9.9×10^{-1}
ERBB2_234354_x_at	1	1.0×10^{0}
SRC_221284_s_at	1	9.9×10^{-1}
FYN_216033_s_at	0.99	9.5×10^{-1}
SYK_209269_s_at	0.99	9.4×10^{-1}
ERBB3_1563252_at	0.99	9.3×10^{-1}
JAK3_211108_s_at	0.99	9.2×10^{-1}
ERBB1_210984_x_at	0.99	9.1×10^{-1}
ERBB3_215638_at	0.99	8.6×10^{-1}
ERBB1_201983_s_at	0.99	8.7×10^{-1}
JAK2_205841_at	0.98	8.5×10^{-1}
ERBB1_201984_s_at	0.98	8.4×10^{-1}
SRC_1565080_at	0.98	8.4×10^{-1}
BLK_210934_at	0.98	7.9×10^{-1}
ERBB1_211550_at	0.98	8.1×10^{-1}
ERBB1_211551_at	0.98	8.1×10^{-1}
JAK3_207187_at	0.98	7.4×10^{-1}
JAK3_211109_at	0.98	7.2×10^{-1}
ERBB1_1565484_x_at	0.97	7.1×10^{-1}
ERBB2_216836_s_at	0.96	6.0×10^{-1}
JAK3_227677_at	0.95	4.9×10^{-1}
JAK1_240613_at	0.95	4.2×10^{-1}
JAK1_1552610_a_at	0.94	3.6×10^{-1}
SYK_244023_at	0.93	3.0×10^{-1}
JAK1_239695_at	0.93	2.7×10^{-1}
SYK_207540_s_at	0.92	2.3×10^{-1}
JAK1_201648_at	0.91	1.5×10^{-1}
PTK2_241453_at	0.9	1.3×10^{-1}
LCK_204890_s_at	0.9	1.9×10^{-1}
ERBB1_1565483_at	0.9	1.9×10^{-1}
PTK2_1559529_at	0.87	3.3×10^{-2}
BLK_206255_at	0.81	2.1×10^{-3}
LYN_202625_at	0.81	9.7×10^{-3}
PTK2_207821_s_at	0.79	6.6×10^{-4}
LYN_202626_s_at	0.75	4.6×10^{-4}
LYN_210754_s_at	0.75	3.7×10^{-4}
FGR_208438_s_at	0.74	3.4×10^{-4}
LCK_204891_s_at	0.73	1.0×10^{-4}
PTK2_208820_at	0.59	$<1 \times 10^{-8}$

Table S13. Gene Expression Levels for Tyrosine Kinases in Leukemic Cells from Pediatric AML Patients with KMT2A/MLL-R$^+$ vs. Pediatric AML Patients with FLT3-ITD$^+$ mutation.

Probeset	Fold Change (Ped AML MLL-R$^+$ / FLT3-ITD$^+$)	Linear Contrast (P-value)
FGR_208438_s_at	4.40	$<1 \times 10^{-8}$
SYK_207540_s_at	3.81	$<1 \times 10^{-8}$
JAK1_1552611_a_at	2.78	$<1 \times 10^{-8}$
SYK_226068_at	2.75	$<1 \times 10^{-8}$
HCK_208018_s_at	2.69	$<1 \times 10^{-8}$
PTK2_208820_at	2.44	$<1 \times 10^{-8}$
JAK2_205841_at	2.20	$<1 \times 10^{-8}$
SYK_209269_s_at	2.16	$<1 \times 10^{-8}$
LYN_210754_s_at	2.13	$<1 \times 10^{-8}$
LYN_202626_s_at	2.10	$<1 \times 10^{-8}$
LCK_204891_s_at	2.10	$<1 \times 10^{-8}$
JAK2_205842_s_at	2.02	$<1 \times 10^{-8}$
LYN_202625_at	2.00	$<1 \times 10^{-8}$
JAK1_239695_at	1.83	$<1 \times 10^{-8}$
FYN_212486_s_at	1.56	1.0×10^{-5}
JAK1_201648_at	1.53	2.4×10^{-5}
BLK_206255_at	1.53	2.5×10^{-5}
JAK2_1562031_at	1.46	1.5×10^{-4}
TYK2_205546_s_at	1.39	1.1×10^{-3}
LCK_204890_s_at	1.39	1.2×10^{-3}
SYK_244023_at	1.38	1.5×10^{-3}
FYN_210105_s_at	1.31	8.1×10^{-3}
JAK1_1552610_a_at	1.30	8.8×10^{-3}
PTK2_1559529_at	1.25	2.6×10^{-2}
PTK2_207821_s_at	1.21	5.5×10^{-2}
JAK1_240613_at	1.18	9.8×10^{-2}
BTK_205504_at	1.15	1.6×10^{-1}
JAK3_227677_at	1.15	1.8×10^{-1}
MERTK_206028_s_at	1.14	2.0×10^{-1}
PTK2_241453_at	1.13	2.2×10^{-1}
MERTK_211913_s_at	1.07	5.0×10^{-1}
FYN_216033_s_at	1.05	6.0×10^{-1}
SRC_221284_s_at	1.05	6.2×10^{-1}
SRC_213324_at	1.03	7.4×10^{-1}
BMX_206464_at	1.03	8.0×10^{-1}
ERBB2_234354_x_at	1.01	9.3×10^{-1}
JAK3_211109_at	1	9.9×10^{-1}
SRC_1558211_s_at	0.98	8.4×10^{-1}
ERBB2_210930_s_at	0.98	8.1×10^{-1}
ERBB2_216836_s_at	0.96	7.1×10^{-1}
JAK3_211108_s_at	0.96	6.9×10^{-1}
BLK_210934_at	0.96	6.7×10^{-1}
ERBB3_1563253_s_at	0.96	6.7×10^{-1}
SRC_1558210_at	0.95	6.0×10^{-1}
JAK3_207187_at	0.93	4.9×10^{-1}
ERBB3_1563252_at	0.93	4.7×10^{-1}
ERBB1_211551_at	0.91	3.3×10^{-1}
SRC_221281_at	0.89	2.6×10^{-1}
ERBB1_224999_at	0.89	2.4×10^{-1}
ERBB1_201984_s_at	0.87	1.6×10^{-1}
ERBB1_211550_at	0.86	1.5×10^{-1}
ERBB3_215638_at	0.86	1.3×10^{-1}
ERBB3_202454_s_at	0.86	1.2×10^{-1}
TEC_206301_at	0.85	1.0×10^{-1}
FLT3_206674_at	0.84	8.8×10^{-2}
ERBB3_226213_at	0.83	7.0×10^{-2}
SRC_1565082_x_at	0.83	6.5×10^{-2}
ERBB1_201983_s_at	0.81	3.9×10^{-2}
ERBB1_210984_x_at	0.80	2.7×10^{-2}
SRC_1565080_at	0.77	1.0×10^{-2}
ERBB1_211607_x_at	0.75	4.9×10^{-3}
MERTK_211912_at	0.73	1.6×10^{-3}
ERBB1_1565484_x_at	0.57	2.5×10^{-8}
ERBB1_1565483_at	0.56	$<1 \times 10^{-8}$

Table S14. Gene Expression Levels for Tyrosine Kinases in Leukemic Cells from Pediatric Patients with KMT2A/MLL-R$^+$ AML vs. Pediatric AML patients with FLT3-ITD$^+$, NPM1$^+$ or CEBPA$^+$.

Probeset	Fold Change (Ped AML MLL-R$^+$ / NPM1$^+$, CEBPA$^+$, FLT3-ITD$^+$)	Linear Contrast (P-value)
FGR_208438_s_at	4.31	$<1 \times 10^{-8}$
SYK_207540_s_at	3.92	$<1 \times 10^{-8}$
HCK_208018_s_at	2.86	$<1 \times 10^{-8}$
SYK_226068_at	2.85	$<1 \times 10^{-8}$
JAK1_1552611_a_at	2.78	$<1 \times 10^{-8}$
PTK2_208820_at	2.46	$<1 \times 10^{-8}$
SYK_209269_s_at	2.19	$<1 \times 10^{-8}$
JAK2_205841_at	2.17	$<1 \times 10^{-8}$
JAK2_205842_s_at	2.04	$<1 \times 10^{-8}$
LYN_210754_s_at	1.96	$<1 \times 10^{-8}$
LYN_202626_s_at	1.93	$<1 \times 10^{-8}$
LCK_204891_s_at	1.9	$<1 \times 10^{-8}$
LYN_202625_at	1.86	$<1 \times 10^{-8}$
JAK1_239695_at	1.79	$<1 \times 10^{-8}$
FYN_212486_s_at	1.54	6.0×10^{-7}
BLK_206255_at	1.51	2.3×10^{-6}
JAK2_1562031_at	1.49	4.3×10^{-6}
JAK1_201648_at	1.44	2.6×10^{-5}
TYK2_205546_s_at	1.41	6.1×10^{-5}
LCK_204890_s_at	1.36	3.3×10^{-4}
SYK_244023_at	1.3	2.3×10^{-3}
BTK_205504_at	1.27	5.2×10^{-3}
JAK1_1552610_a_at	1.26	7.6×10^{-3}
PTK2_1559529_at	1.26	8.3×10^{-3}
FYN_210105_s_at	1.25	9.1×10^{-3}
PTK2_207821_s_at	1.2	3.3×10^{-2}
JAK1_240613_at	1.17	6.8×10^{-2}
PTK2_241453_at	1.12	2.0×10^{-1}
MERTK_206028_s_at	1.1	2.7×10^{-1}
SRC_221284_s_at	1.08	3.6×10^{-1}
FLT3_206674_at	1.08	3.8×10^{-1}
MERTK_211913_s_at	1.08	3.8×10^{-1}
JAK3_227677_at	1.06	5.2×10^{-1}
BMX_206464_at	1.04	6.6×10^{-1}
SRC_213324_at	1.04	6.6×10^{-1}
FYN_216033_s_at	1.03	7.6×10^{-1}
ERBB2_234354_x_at	1.01	8.7×10^{-1}
JAK3_211109_at	1	9.7×10^{-1}
SRC_1558211_s_at	1	9.8×10^{-1}
ERBB2_210930_s_at	0.99	9.4×10^{-1}
ERBB3_1563253_s_at	0.97	6.9×10^{-1}
JAK3_211108_s_at	0.96	6.8×10^{-1}
SRC_1558210_at	0.96	6.6×10^{-1}
BLK_210934_at	0.96	6.1×10^{-1}
ERBB2_216836_s_at	0.95	5.9×10^{-1}
ERBB3_1563252_at	0.94	4.8×10^{-1}
JAK3_207187_at	0.93	4.2×10^{-1}
SRC_221281_at	0.91	2.5×10^{-1}
ERBB1_211551_at	0.9	2.1×10^{-1}
ERBB1_224999_at	0.89	1.8×10^{-1}
TEC_206301_at	0.88	1.6×10^{-1}
ERBB3_215638_at	0.86	8.9×10^{-2}
ERBB3_202454_s_at	0.86	8.7×10^{-2}
ERBB1_211550_at	0.86	8.5×10^{-2}
SRC_1565082_x_at	0.85	6.6×10^{-2}
ERBB1_201984_s_at	0.85	6.2×10^{-2}
ERBB3_226213_at	0.84	4.4×10^{-2}
ERBB1_210984_x_at	0.8	8.9×10^{-3}
ERBB1_201983_s_at	0.79	7.3×10^{-3}
SRC_1565080_at	0.77	2.8×10^{-3}
ERBB1_211607_x_at	0.76	1.2×10^{-3}
MERTK_211912_at	0.72	1.5×10^{-4}
ERBB1_1565484_x_at	0.56	$<1 \times 10^{-8}$
ERBB1_1565483_at	0.55	$<1 \times 10^{-8}$
